# *Setd2* overexpression rescues bivalent gene expression during SCNT-mediated ZGA

**DOI:** 10.1093/procel/pwaf010

**Published:** 2025-02-13

**Authors:** Xiaolei Zhang, Ruimin Xu, Yuyan Zhao, Yijia Yang, Qi Shi, Hong Wang, Xiaoyu Liu, Shaorong Gao, Chong Li

**Affiliations:** Shanghai Key Laboratory of Maternal Fetal Medicine, Shanghai Institute of Maternal-Fetal Medicine and Gynecologic Oncology, Clinical and Translation Research Center, Shanghai First Maternity and Infant Hospital, School of Life Sciences and Technology, Tongji University, Shanghai 200092, China; State Key Laboratory of Cardiology and Medical Innovation Center, Shanghai East Hospital, School of Life Sciences and Technology, Tongji University, Shanghai 200092, China; Shanghai Key Laboratory of Maternal Fetal Medicine, Shanghai Institute of Maternal-Fetal Medicine and Gynecologic Oncology, Clinical and Translation Research Center, Shanghai First Maternity and Infant Hospital, School of Life Sciences and Technology, Tongji University, Shanghai 200092, China; State Key Laboratory of Cardiology and Medical Innovation Center, Shanghai East Hospital, School of Life Sciences and Technology, Tongji University, Shanghai 200092, China; Shanghai Key Laboratory of Maternal Fetal Medicine, Shanghai Institute of Maternal-Fetal Medicine and Gynecologic Oncology, Clinical and Translation Research Center, Shanghai First Maternity and Infant Hospital, School of Life Sciences and Technology, Tongji University, Shanghai 200092, China; State Key Laboratory of Cardiology and Medical Innovation Center, Shanghai East Hospital, School of Life Sciences and Technology, Tongji University, Shanghai 200092, China; Shanghai Key Laboratory of Maternal Fetal Medicine, Shanghai Institute of Maternal-Fetal Medicine and Gynecologic Oncology, Clinical and Translation Research Center, Shanghai First Maternity and Infant Hospital, School of Life Sciences and Technology, Tongji University, Shanghai 200092, China; State Key Laboratory of Cardiology and Medical Innovation Center, Shanghai East Hospital, School of Life Sciences and Technology, Tongji University, Shanghai 200092, China; Shanghai Key Laboratory of Maternal Fetal Medicine, Shanghai Institute of Maternal-Fetal Medicine and Gynecologic Oncology, Clinical and Translation Research Center, Shanghai First Maternity and Infant Hospital, School of Life Sciences and Technology, Tongji University, Shanghai 200092, China; State Key Laboratory of Cardiology and Medical Innovation Center, Shanghai East Hospital, School of Life Sciences and Technology, Tongji University, Shanghai 200092, China; Shanghai Key Laboratory of Maternal Fetal Medicine, Shanghai Institute of Maternal-Fetal Medicine and Gynecologic Oncology, Clinical and Translation Research Center, Shanghai First Maternity and Infant Hospital, School of Life Sciences and Technology, Tongji University, Shanghai 200092, China; Shanghai Key Laboratory of Maternal Fetal Medicine, Shanghai Institute of Maternal-Fetal Medicine and Gynecologic Oncology, Clinical and Translation Research Center, Shanghai First Maternity and Infant Hospital, School of Life Sciences and Technology, Tongji University, Shanghai 200092, China; State Key Laboratory of Cardiology and Medical Innovation Center, Shanghai East Hospital, School of Life Sciences and Technology, Tongji University, Shanghai 200092, China; Frontier Science Center for Stem Cell Research, Tongji University, Shanghai 200092, China; Shanghai Key Laboratory of Maternal Fetal Medicine, Shanghai Institute of Maternal-Fetal Medicine and Gynecologic Oncology, Clinical and Translation Research Center, Shanghai First Maternity and Infant Hospital, School of Life Sciences and Technology, Tongji University, Shanghai 200092, China; State Key Laboratory of Cardiology and Medical Innovation Center, Shanghai East Hospital, School of Life Sciences and Technology, Tongji University, Shanghai 200092, China; Frontier Science Center for Stem Cell Research, Tongji University, Shanghai 200092, China; Shanghai Key Laboratory of Maternal Fetal Medicine, Shanghai Institute of Maternal-Fetal Medicine and Gynecologic Oncology, Clinical and Translation Research Center, Shanghai First Maternity and Infant Hospital, School of Life Sciences and Technology, Tongji University, Shanghai 200092, China; Frontier Science Center for Stem Cell Research, Tongji University, Shanghai 200092, China

**Keywords:** histone modifications, SCNT, embryo development

## Abstract

Successful cloning through somatic cell nuclear transfer (SCNT) faces significant challenges due to epigenetic obstacles. Recent studies have highlighted the roles of H3K4me3 and H3K27me3 as potential contributors to these obstacles. However, the underlying mechanisms remain largely unclear. In this study, we generated genome-wide maps of H3K4me3 and H3K27me3 in mouse pre-implantation NT embryos. Our analysis revealed that aberrantly over-represented broad H3K4me3 domain and H3K27me3 signal lead to increased bivalent marks at gene promoters in NT embryos compared with naturally fertilized (NF) embryos at the 2-cell stage, which may link to relatively low levels of H3K36me3 in NT 2-cell embryos. Notably, the overexpression of *Setd2*, a H3K36me3 methyltransferase, successfully restored multiple epigenetic marks, including H3K36me3, H3K4me3, and H3K27me3. In addition, it reinstated the expression levels of ZGA-related genes by reestablishing H3K36me3 at gene body regions, which excluded H3K27me3 from bivalent promoters, ultimately improving cloning efficiency. These findings highlight the excessive bivalent state at gene promoters as a potent barrier and emphasize the removal of these barriers as a promising approach for achieving higher cloning efficiency.

## Introduction

Somatic cell nuclear transfer (SCNT) empowers terminally differentiated somatic cells to attain totipotency (Lu and Zhang, 2015), enabling the generation of cloned animals and nuclear transfer embryonic stem cells (ntESCs) (Tachibana et al., 2013; Wakayama et al., 2001; Zhang et al., 2021). This technology holds promise not only for the conservation of endangered species but also for potential therapeutic applications in humans (Chung et al., 2014, 2015; Yamada et al., 2014). Despite these potential advancements, the practical implementation of SCNT technology faces significant challenges, primarily due to the low efficiency of SCNT-mediated reprogramming, coupled with the occurrence of defects in extraembryonic tissues and abnormalities in cloned individuals (Matoba and Zhang, 2018; Zhang et al., 2021). Abnormalities in cloned embryos, such as aberrant DNA methylation, compromised histone modifications, and incomplete zygotic genome activation (ZGA) (Teperek and Miyamoto, 2013; Zhang et al., 2021), underscore the inherent challenges associated with epigenetic reprogramming (Chen et al., 2020; Gao et al., 2018; Kang et al., 2014; Matoba et al., 2018). Recognizing that the process of epigenetic reprogramming is susceptible to unavoidable failures that impede NT embryo development, a comprehensive understanding of the epigenetic reprogramming process is indispensable for prompting the improvement of SCNT technology.

H3K4me3 and H3K27me3 are pivotal epigenetic regulators well known for their roles as a transcriptional activator and repressor, respectively (Millán-Zambrano et al., 2022; Simon and Kingston, 2009). The proper reprogramming of these two markers is crucial for the successful development of mammalian embryos (Xu et al., 2020; Zheng et al., 2016). Previous studies have demonstrated the significance of the switch from non-canonical H3K4me3 (ncH3K4me3) patterns in oocytes to canonical patterns in 2-cell stage embryos for mouse early embryo development (Bu et al., 2022; Zhang et al., 2016). As the embryo develops, the canonical H3K4me3 domains gradually broaden, forming distinct enriched broad H3K4me3 domains that differ from the ncH3K4me3 domains. These broad domains are associated with higher transcriptional activity during pre-implantation development. Downregulating of H3K4me3 demethylases compromises ZGA and impairs embryo development (Dahl et al., 2016; Liu et al., 2016b; Zhang et al., 2016). Also, reprogramming defects of H3K4me3 in NT embryos have been identified. In our previous work, we uncovered that the deficiency of *Kdm5b*, a demethylase of H3K4me3, in NT embryos, led to 4-cell arrest. Overexpression of *Kdm5b* assisted NT embryos in overcoming this arrest and restored the expression levels of genes in NT 4-cell arrest samples (Liu et al., 2016a). Consistent with our findings, another investigation reported that the removing donor-inherited H3K4me3 substantially improved transcriptional reprogramming and development in *Xenopus* NT embryos (Hörmanseder et al., 2017). These findings suggest that H3K4me3 may act as a barrier during SCNT-mediated reprogramming.

In addition to the transcriptionally active marker, the repressor H3K27me3 has also been extensively studied for its critical role in embryogenesis (Inoue et al., 2018; Liu et al., 2016b; Zhang et al., 2019; Zheng et al., 2016). Aberrant reprogramming of H3K27me3 poses a significant hurdle in the development of NT embryos across various species (Matoba et al., 2018; Xie et al., 2016; Zhou et al., 2019). Numerous genes exhibit allele-specific H3K27me3, including *Sfmbt2*, *Jade1*, *Gab1* and *Smoc1* (Inoue et al., 2017; Matoba et al., 2018). The loss of H3K27me3 imprinting in NT embryos disrupts mouse post-implantation development (Inoue et al., 2020; Matoba et al., 2018). The correction of the expression of clustered miRNAs within the *Sfmbt2* gene and the quadruple monoallelic deletion of *Sfmbt2*, *Jade1*, *Gab1,* and *Smoc1* have been demonstrated to ameliorate the abnormal placental phenotype (Inoue et al., 2020; Wang et al., 2020). Consistently, another approach involved the overexpression of an H3K27me3-specific demethylase, KDM6A, which significantly improved cloning efficiency (Yang et al., 2018; Zhou et al., 2019). In contrast, the knockdown of KDM6B, another H3K27me3-specific demethylase, not only facilitated ZGA but also enhanced cloning efficiency and ntESC establishment efficiency (Yang et al., 2018). Therefore, both the proper deposition on specific regions (such as H3K27me3-imprinted genes) and the appropriate removal of H3K27me3 are crucial for the successful reprogramming of NT embryos.

However, it remains largely unclear how H3K4me3 and H3K27me3 are reprogrammed during the early development of NT embryos. In this study, we characterized the landscapes of H3K4me3 and H3K27me3 during pre-implantation mouse NT embryogenesis. Comparative analysis with naturally fertilized (NF) embryos uncovered aberrant over-representation of both H3K4me3 and H3K27me3 in 2-cell stage NT embryos, leading to severe expression defects in ZGA genes with a bivalent state. Given the established knowledge of the mutually exclusive state of H3K36me3 with H3K4me3/H3K27me3 (Xu et al., 2019), we overexpressed *Setd2*, a H3K36me3-specific methyltransferase, in NT embryos. We found that overexpression of *Setd2* successfully rescued ZGA gene expression in NT embryos, allowing these genes to exit the bivalent state by enhancing H3K36me3 at gene bodies and excluding H3K27me3 from promoters. Overall, our study revealed the mechanisms underlying the aberrant reprogramming of H3K4me3 and H3K27me3 and successfully rescued the reprogramming defects, eventually improving cloning efficiency.

## Results

### Reprogramming of H3K4me3 and H3K27me3 during mouse SCNT embryogenesis

To elucidate the genome-wide landscape of H3K4me3 and H3K27me3 modifications during mouse somatic cell nuclear transfer (SCNT) embryogenesis, we conducted ultra-low-input chromatin immunoprecipitation followed by sequencing (ULI-NChIP-seq) (Brind’Amour et al., 2015) in reconstructed embryos at various stages, including 6 h post-activation (hpa), 14 hpa, 2-cell, 4-cell, 8-cell, morula, as well as inner cell mass (ICM) and trophectoderm (TE) from blastocysts and cumulus cells (CCs), which served as donor cells for SCNT (Fig. S1A; Table S1). All replicates exhibited high reproducibility (Fig. S1B–D; Table S2).

**Figure 1.**
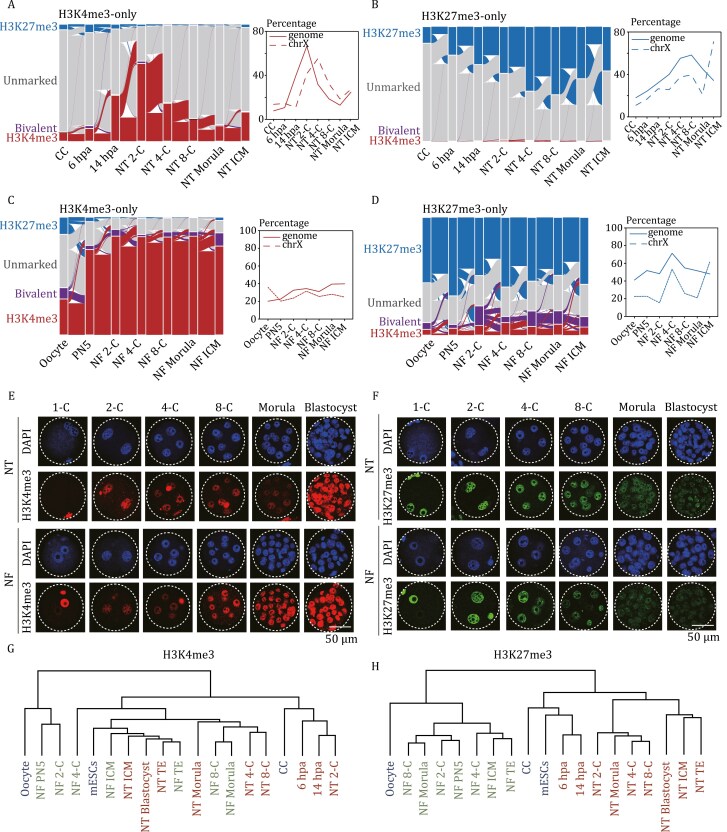
**Dynamics of genome-wide H3K4me3 and H3K27me3 landscapes in mouse SCNT pre-implantation embryos.** (A and B) Alluvial plots (left panel) depict the global dynamics of H3K4me3-only (A) and H3K27me3-only (B) regions during NT early embryo development. Each line represents a 200 bp bin defined on the ChromHMM categories. The 200 bp bins marked as H3K4me3-only (A) and H3K27me3-only (B) at all developmental stages were extracted. Chromatin states of these bins throughout the entire developmental process were presented. The global dynamics (solid line) and chromosome X dynamics (dashed line) are plotted separately (right panel). (C and D) Alluvial plots (left panel) depict the global dynamics of H3K4me3-only (C) and H3K27me3-only (D) regions during NF early embryo development. Each line represents a 200 bp bin defined on the ChromHMM categories. The 200 bp bins marked as H3K4me3-only (C) and H3K27me3-only (D) at all developmental stages were extracted. Chromatin states of these bins throughout the entire developmental process were presented. The global dynamics (solid line) and chromosome X dynamics (dashed line) are plotted separately (right panel). (E and F) Immunostaining of H3K4me3 (E) and H3K27me3 (F) in each stage of NF and NT pre-implantation embryos. DAPI stains for DNA. A representative image from three independent experiments is presented. Scale bar: 50 μm. (G and H) Hierarchical clustering of individual stages of nuclear transfer (NT) and natural fertilized (NF) embryogenesis, along with cell lines CCs, oocyte and mouse embryonic stem cells (mESCs), based on global H3K4me3 (G) and H3K27me3 (H) enrichment.

To characterize the chromatin state in pre-implantation nuclear transfer (NT) and natural fertilized (NF) embryos, we employed ChromHMM analysis to categorize the genome into four chromatin states based on the profiles of H3K4me3 and H3K27me3 modifications at each stage, which are H3K4me3-only regions, H3K27me3-only regions, bivalent regions, and unmarked regions (Figs. 1A–D and S2A–E). We divided the genome into bins of 200 bp each. The classification of each bin was determined based on the ratio of the signal to the input within these 200 bp regions. Bins marked by both H3K4me3 and H3K27me3 were defined as “Bivalent”. Bins marked only by H3K4me3 were classified as “H3K4me3-only,” while those marked only by H3K27me3 were defined as “H3K27me3-only.” Bins not marked by either H3K4me3 or H3K27me were classified as “Unmarked.” Consistent with immunofluorescence staining (Figs. 1E and S2F), ULI-NChIP-seq data revealed that global H3K4me3-only regions accumulate progressively after activation, peaking at 2-cell stage, and gradually decreasing until the ICM, with a slight increase at this stage (Fig. 1A). Similarly, the global number of H3K27me3-only regions follows a gradual increase and decrease pattern during NT embryo development (Figs. 1B, 1F and S2G). Bivalent regions exhibited more variable dynamics throughout the entire early embryogenesis in both NF and NT embryos (Fig. S2A and S2D).

We also performed immunostaining for H3K4me3 and H3K27me3 in naturally fertilized (NF) pre-implantation embryos. The results showed discrepancies of H3K4me3 and H3K27me3 signals between NT and NF embryos at the same developmental stages (Figs. 1E, 1F, S2F and S2G), indicating distinct reprogramming progress of H3K4me3 and H3K27me3 between NT and NF embryos. Furthermore, we explored potential differences in H3K4me3 and H3K27me3 modification patterns between SCNT and fertilized embryo development using sequencing data. As expected, hierarchical clustering revealed genome-wide differences in H3K4me3 dynamics between NT and NF embryos at the 2-cell and earlier stages (Fig. 1G). In contrast, genome-wide differences in H3K27me3 dynamics between NT and NF embryos were evident throughout all stages of early embryogenesis (Fig. 1H). Moreover, while the distribution of H3K4me3 showed minimal differences between NT and NF embryos at corresponding stages (Fig. S3A), there was a significant dissimilarity in H3K27me3 modification coverage on distinct genome features between NT and NF embryos, particularly at promoter regions and retrotransposons (Fig. S3B), suggesting a unique reprogramming progression in NT embryos. These differences likely arise from distinct expression levels of writers and erasers of H3K4me3 and H3K27me3 modifications during NT and NF embryogenesis (Fig. S3C and S3D).

### Aberrantly broadened H3K4me3 domains are related to impaired gene expression in NT embryos

**Figure 2.**
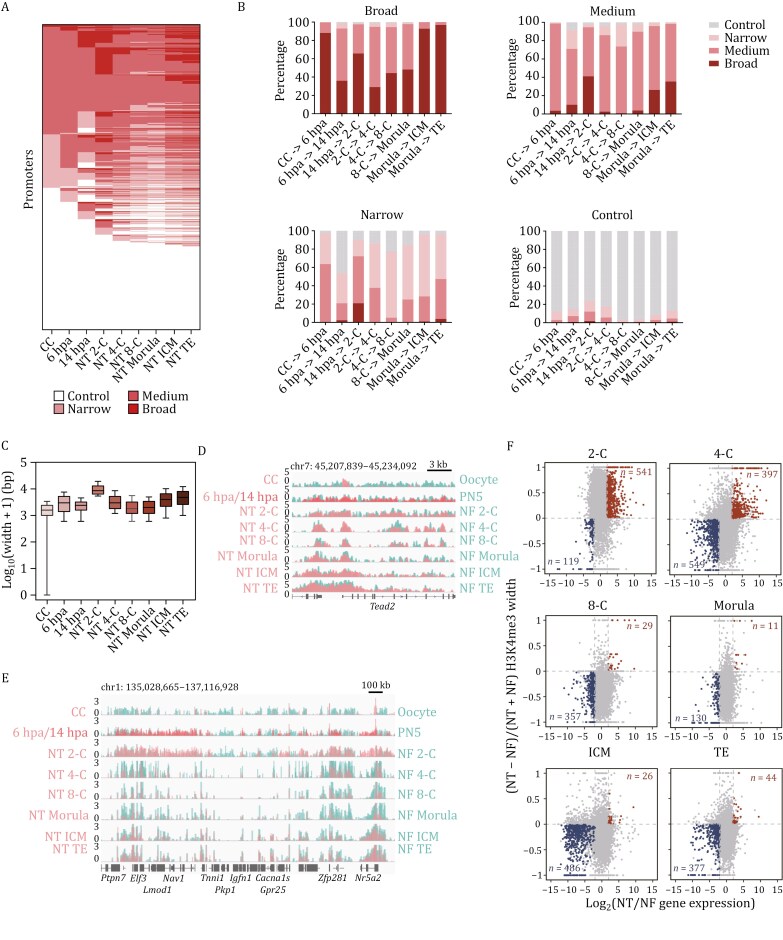
**Aberrantly broadened H3K4me3 domains are related to impaired gene expression in NT embryos.** (A) Dynamics of the H3K4me3 domain width on all RefSeq gene promoters throughout NT embryo development. Promoters are classified based on breadth of marked H3K4me3 domains: broad, medium, narrow and control. (B) Tendencies of the four types of H3K4me3 domains in promoters in the next stage during NT embryogenesis. Each panel represents a specific type of H3K4me3 domain as marked above. Each bar represents the types and fractions of promoters in the next stage. (C) Bar chart showing the averaged H3K4me3 domain width [log_10_(width + 1) bp] of all RefSeq gene promoters in each stage of NT embryos and donor cells, CCs. Data represented as mean ± SD. (D) Genome browser view of H3K4me3 signal near *Tead2* promoter region throughout NT and NF embryo development. H3K4me3 enrichment was calculated as log_2_(H3K4me3 RPKM/input RPKM). (E) Genome browser view of H3K4me3 signal on a random region of chromosome 1 during NT and NF embryogenesis. H3K4me3 enrichment was calculated as log_2_(H3K4me3 RPKM/input RPKM). (F) Scatterplots showing the comparison between gene expression and H3K4me3 domain width at relative gene promoter at specific stages of NT versus NF embryos. Each point represents a RefSeq gene. Genes up-regulated in NT embryos compared to correlated NF embryos [log_2_FC > 2, *P.adj*. < 0.01, FC: fold change] with enlarged H3K4me3 domain width are plotted as red, defined as obH3K4me3 genes (genes overexpressed with broadened H3K4me3 domains at their promoter regions). Genes down-regulated in NT embryos compared to correlated NF embryos [log_2_FC < −2, *P.adj*. < 0.01] with shortened H3K4me3 domain width are plotted as blue, defined as usH3K4me3 genes (genes under-expressed with shortened H3K4me3 domains at their promoter regions).

Given that H3K4me3 is commonly regarded as a positive marker associated with transcription initiation (Huang et al., 2019; Wang et al., 2023), our initial focus was on understanding the dynamics of H3K4me3 modification at promoter regions. Previous studies have shown that broad H3K4me3 domains (wider than 5 kb) are linked to higher transcriptional activity during pre-implantation development, and are preferentially enriched in genes essential for the function and identity of a given cell type (Benayoun et al., 2014; Chen et al., 2015; Liu et al., 2016b). To dissect the details of H3K4me3 breadth dynamics during SCNT-mediated reprogramming and delineate differences in this dynamic between NT and NF embryos, we categorized H3K4me3 domains at each stage around transcription start sites (TSSs) into three groups: broad domains (exceeding 5 kb), medium domains (approximately 1–5 kb), and narrow domains (less than 1 kb), those less than 200 bp were defined as controls, which are considered as not marked by H3K4me3 (Figs. 2A and S4A; Table S3). Surprisingly, while the total number of genes marked by H3K4me3 exhibited minimal fluctuation during NT early embryogenesis, numerous broad H3K4me3 domains were dramatically established at the 2-cell stage, followed by a rapid decrease until the blastocyst stage (Figs. 2A and S4B). This pattern sharply contrasts with the gradual establishment observed in fertilized embryos (Fig. S4A and S4C) (Liu et al., 2016b). The high dynamic of H3K4me3 breadth mainly occurred between broad and medium domains across continuous stages, with minimal changes between broad and narrow or control domains (Fig. 2B), akin to the dynamics observed in fertilized embryos (Fig. S4D) (Liu et al., 2016b). This suggests a steady dynamic of H3K4me3 domain breadth. Indeed, a substantial proportion of broad H3K4me3 domains in 2-cell NT embryos enlarged from medium H3K4me3 domains in CCs, then shortened to medium domains in 4-cell stages and beyond, ultimately extending to broad domains in the blastocyst (Fig. 2A). Moreover, the H3K4me3 domain in 2-cell NT embryos exhibited the greatest average breadth throughout early embryogenesis (Fig. 2C), even extending over the entire gene body (Fig. 2D and 2E). Expectedly, when comparing the average length and distribution of H3K4me3 domain length at promoter regions between corresponding stages of NT and NF embryos, we found that the percentage of broad H3K4me3 domains appears to be increased in NT compared to NF embryos at 2-C stage, while it appears to be decreased at the 4-C, 8-C and morula stages (Fig. S4E and S4F), indicating a distinct H3K4me3 reprogramming pattern during NT embryogenesis (Fig. S4G).

Considering that H3K4me3 domain breadth is correlated with gene expression in NT embryos (Fig. S5A), consistent with NF embryos (Liu et al., 2016b), we wondered whether the discrepancy in H3K4me3 domain length affected gene expression patterns in NT embryos. We aimed to identify genes affected by aberrant H3K4me3 by comparing changes in expression levels and H3K4me3 width between NT and NF embryos at the same stage (Fig. 2F). RNA-seq data of NT and NF embryos are from GSE195762 and GSE71434, and H3K4me3 ChIP-seq data of NF embryos are from GSE73952. Genes up-regulated in NT embryos with promoters marked by broader H3K4me3 domains compared to NF embryos at the same stage were defined as over-expressed with broadened H3K4me3 (obH3K4me3) genes, while those down-regulated in NT embryos with promoters marked by shorter H3K4me3 domains compared to NF embryos at the same stage were defined as under-expressed with shortened H3K4me3 (usH3K4me3) genes (Fig. 2F). As expected, we identified numerous obH3K4me3 genes (*n* = 541) but few usH3K4me3 genes (*n* = 119) in 2-cell stage NT embryos, highlighting significant expression dysregulation caused by aberrantly broadened H3K4me3 domains. Although there were a substantial number of obH3K4me3 genes (*n* = 397) in 4-cell stage NT embryos, more usH3K4me3 genes (*n* = 549) were defined at the same stage, potentially contributing to the moderate average domain width of H3K4me3 in 4-cell stage NT embryos (Figs. 2C and S4C). Functional analysis of 2-cell obH3K4me3 genes revealed a significant enrichment in metabolic processes, particularly associated with the glutathione metabolic process (Fig. S5B), which is critical for embryo development (Zhao et al., 2021). This hints at the possibility that aberrant reprogramming of H3K4me3 may lead to metabolic disruption in 2-cell stage NT embryos. Thus, our findings demonstrate H3K4me3 domains deposited at these promoter regions presumably contribute to reprogramming defects and disrupted metabolism by dysregulating gene expression in NT embryos.

### Shortening H3K4me3 domain breadth with WDR5-0103 improves cloning efficiency

**Figure 3.**
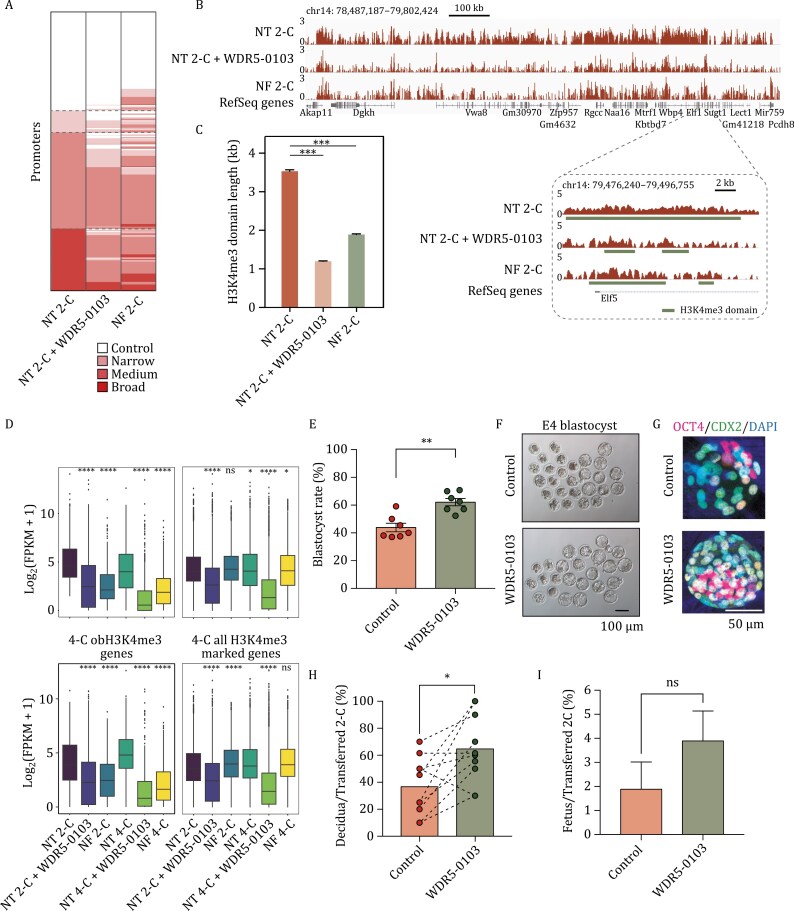
**Shortening the length of H3K4me3 domains in 2-cell stage NT embryos improves cloning efficiency.** (A) Heatmap displays the categories of H3K4me3 domains on promoters in nuclear transfer 2-cell stage embryos (NT 2-C), nuclear transfer 2-cell stage embryos with WDR5-0103 treatment (NT 2-C + WDR5-0103) and natural fertilized 2-cell stage embryos (NF 2-C). H3K4me3 domains classified into four groups: broad domains, medium domains, narrow domains, and control domains. Dashed lines represent classification based on NT 2-C sample. (B) Genome browser view shows H3K4me3 enrichment of NT 2-C, NT 2-C + WDR5-0103 and NF 2-C samples at a random region of chromosome 14. The dashed box displays the genome browser view of H3K4me3 enrichment on *Elf5* gene locus. H3K4me3 enrichment calculated as log_2_(H3K4me3 RPKM / input RPKM). (C) Boxplot illustrates the averaged breadth of H3K4me3 domain at all RefSeq gene promoters in NT 2-C, NT 2-C + WDR5-0103, and NF 2-C samples. Data represented as the mean ± SD. Significance analyzed using Student’s *t*-test (***P < 0.001). (D) Averaged expression level [log_2_(FPKM + 1)] of 2-C (left panel) and 4-C (right panel) obH3K4me3 genes and all H3K4me3 marked genes in NT 2-C, NT 2-C + WDR5-0103, NF 2-C, NT 4-C, NT 4-C + WDR5-0103 and NF 4-C samples. Significance analyzed by using Student’s *t*-test (ns: not significant, *P < 0.05, ****P < 0.0001). (E) Bar chart shows the percentage of embryos reaching blastocyst stage for NT (control) and NT + WDR5-0103 (WDR5-0103) samples. Data represented as mean ± SD (*n* = 7). Significance analyzed using Student’s *t*-test (**P < 0.01). (F) Representative images of E4.5 blastocyst of NT (control) and NT + WDR5-0103 (WDR5-0103) samples. (G) Immunostaining of OCT4 and CDX2 in control NT E4 blastocysts (control) and NT E4 blastocysts with WDR5-0103 treatment at the 2-cell stage (WDR5-0103). DAPI stains for DNA (blue), OCT4 is visualized in pink and CDX2 is visualized in green. Scale bar: 50 μm. (H) Bar chart shows the percentage of implantation rate of NT (control) and NT + WDR5-0103 (WDR5-0103) samples examined by cesarean section on E19.5. Each dashed line links a parallel experiment of control and WDR5-0103 sample. Data represented as mean ± SD (*n* = 7). Significance analyzed using Student’s *t*-test (*P < 0.05). (I) Bar chart displaying birth rates of NT embryos without WDR5-0103 treatment (control) and with WDR5-0103 treatment at 2-cell stage (WDR5-0103). Birth rates are calculated as the number of fetuses divided by the number of 2-cells transferred to recipients. Data are represented as mean ± SD (*n ≥* 3). Significance was analyzed by using Student’s *t*-test (ns: not significant).

Given the large-scale abnormal establishment of H3K4me3 during the 2-cell stage of NT embryos, we introduced WDR5-0103, a WDR5 antagonist that disrupts WDR5 interaction with MLL and inhibits MLL core complex methyltransferase activity, into NT embryos post-activation, with the aim of preventing H3K4me3 establishment (Fig. S6A). To assess whether the addition of WDR5-0103 could successfully rescue H3K4me3 reprogramming defects and subsequently restore gene expression, we conducted ULI-NChIP-seq (Brind’Amour et al., 2015) of H3K4me3 in 2-cell embryos and Smart-seq2 (Picelli et al., 2014) in 2-cell and 4-cell stages of NT embryos following WDR5-0103 treatment (Fig. S6B–D; Tables S1 and S2). As expected, the coverages of H3K4me3 across the whole genome and specific genome regions, such as gene body regions and intergenic regions, were effectively reduced in NT 2-C embryos with WDR5-0103 treatment (NT 2-C + WDR5-0103) compared to NT 2-C embryos without WDR5-0103 treatment (NT 2-C) ( Fig. S6E–G). The distribution of H3K4me3 domain width at promoter regions in 2-cell stage NT embryos after WDR5-0103 treatment resembled those in 2-cell stage NF embryos (Fig. 3A). Moreover, the breadth of H3K4me3 domains at promoter regions was significantly shortened by adding WDR5-0103 compared to NT 2-C embryos without WDR5-0103 treatment (Fig. 3B and 3C). These results suggest that the addition of WDR5-0103 successfully rescued not only overall H3K4me3 coverage but also the specific H3K4me3 domain breadth.

Due to the shortened H3K4me3 domain width near TSSs, the expression levels of obH3K4me3 genes in 2-cell and 4-cell stage NT embryos were statistically downregulated to levels similar to fertilized 2-cell and 4-cell embryos, with the addition of WDR5-0103 (Figs. 3D and S7A). Surprisingly, although expression levels of all H3K4me3-marked genes are barely different between NF and NT embryos at both 2-call and 4-cell stages, WDR5-0103 treatment significantly downregulates the expression levels of these genes in NT embryos compared to NT embryos without WDR5-0103 treatment, similar to the changes observed in obH3K4me3 genes. This implies WDR5-0103 may broadly inhibit the establishment of H3K4me3. Beyond the transcriptome, the blastocyst formation rate for NT embryos were significantly improved by WDR5-0103 treatment (Fig. 3E), and more high-quality blastocysts was observed in WDR5-0103-treated NT embryos (Figs. 3F, 3G and S7B). Furthermore, the implantation rate of NT embryos significantly increased with the addition of WDR5-0103 (Fig. 3H), and the treatment resulted in approximately a two-fold increase in birth rate compared to NT embryos without WDR5-0103 treatment (Fig. 3I). In conclusion, our findings suggest that inhibiting the excessive establishment of H3K4me3, leading to the reduction of H3K4me3 domain breadth in NT embryos, creates a permissive environment for accurate transcriptome content and proper development of NT embryos.

#### Aberrant H3K27me3 impairs expression patterns of genes correlated with embryogenesis

**Figure 4.**
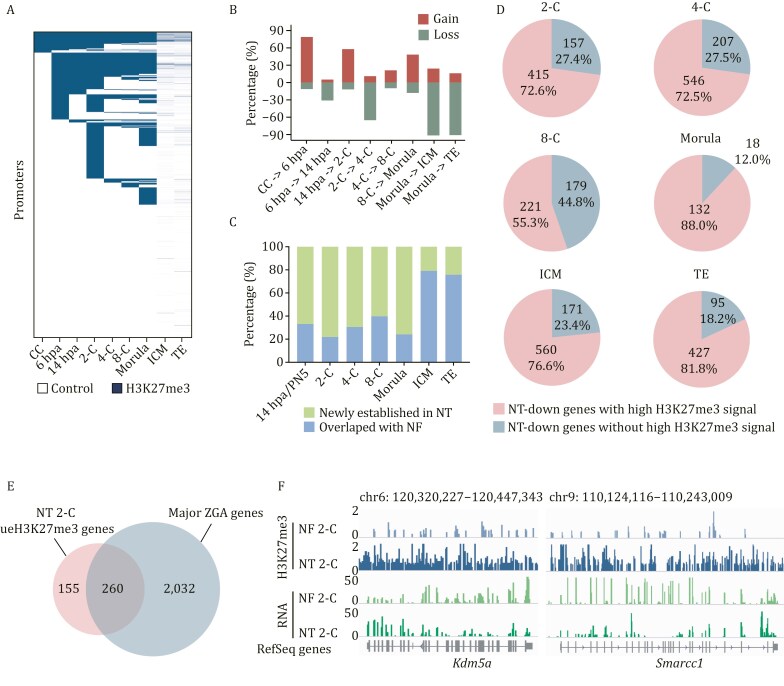
**Over-representation of H3K27me3 at promoters impairs expression patterns of embryogenesis-related genes in NT embryos.** (A) Heatmap displays H3K27me3-marked (H3K27me3) and H3K27me3-unmarked (control) RefSeq gene promoters during NT embryo development. H3K27me3-marked promoters were defined with an overlap of at least 200 bp with ChromHMM H3K27me3 segmentations, including H3K27me3-only and bivalent segmentations. (B) Percentage of RefSeq genes that gaining or losing H3K27me3 marks at their promoter regions in different stages calculated by comparison to the previous stages during NT embryo development. (C) Bar chart shows the percentage of RefSeq genes established with H3K27me3 only in nuclear transfer (NT) embryos and overlapping with natural fertilized (NF) embryos in corresponding embryo development stages. (D) Percentage distribution of NT-downregulated genes with high H3K27me3 signal and NT-downregulated genes without high H3K27me3 signal in each stage of NT embryos. NT-down genes with high H3K27me3 signal represent genes downregulated in NT embryos with enriched H3K27me3 signal at their promoter regions compared with corresponding stages of NF embryos. NT-down genes without high H3K27me3 signal represent genes downregulated in NT embryos without enriched H3K27me3 signal at their promoter regions compared with corresponding stages of NF embryos. (E) Venn plot displays the overlap between 2-C ueH3K27me3 genes and major ZGA genes. (F) Genome browser views depict H3K27me3 enrichment and RNA levels of *Kdm5a* (left panel) and *Smarcc1* (right panel) in NF 2-C and NT 2-C samples.

Based on the aforementioned results, we observed over-enrichment of H3K27me3 at promoter regions in 2-cell stage NT embryos compared to 2-cell stage NF embryos (Fig. S3B), suggesting a potential reprogramming defect of H3K27me3 at promoter regions. Given the concurrent consideration of the repressive impact of H3K27me3 at gene promoters (Di Croce and Helin, 2013; Simon and Kingston, 2009), our primary focus was on investigating the dynamics of H3K27me3 reprogramming at promoter regions and elucidating its implications for gene expression in NT embryos. In comparison to the donor cells, CCs, the number of H3K27me3-marked promoters dramatically increased at the 2-cell stage in NT embryos, displaying dynamic changes throughout development, and then sharply decreased from the morula stage to ICM/TE (Fig. 4A and 4B). This suggests a dynamic reprogramming process during the pre-implantation development of NT embryos. To ascertain whether H3K27me3 reprogramming is impaired in NT embryos compared to NF embryos, we analyzed the percentage of genes marked by H3K27me3 only in NT embryos (Fig. 4C, NT-specific) and those marked simultaneously in NF and NT embryos (Fig. 4C, overlapped with NF) at corresponding stages. Over 60% genes with NT-specific H3K27me3 were defined before blastocyst stage, indicating distinct H3K27me3 signals at promoter regions in NT embryos compared to NF embryos before the blastocyst stage (Fig. 4C). This suggests a potential reprogramming defect of H3K27me3 in NT embryos.

We then investigated the extent to which the reprogramming dysregulation of H3K27me3 affect gene expression patterns in NT embryos. We classified aberrant H3K27me3-affected genes by comparing the changes in expression levels and H3K27me3 enrichment levels on their promoters between NT and NF embryos at corresponding stages (Fig. S8A). Genes downregulated in NT embryos with enriched H3K27me3 signals at their promoter regions compared to NF embryos were defined as under-expressed with enriched H3K27me3 (ueH3K27me3) genes, while genes upregulated in NT embryos with deficient H3K27me3 signals at their promoter regions compared to NF embryos were defined as over-expressed with deficient H3K27me3 (odH3K27me3) genes (Fig. S8A). Interestingly, numerous ueH3K27me3 genes (132–560 genes) were found throughout the entire SCNT-mediated reprogramming progress, accounting for a substantial proportion (55.3%–88.0%) of all down-regulated genes at each stage (Fig. 4D). In contrast, only a few odH3K27me3 genes were identified in NT embryos, possibly due to the generally stronger H3K27me3 signals at promoter regions in NT embryos compared to NF embryos (Fig. S8A). These results indicate that abnormal H3K27me3 may contribute to the aberrant downregulation of genes in NT embryos. To test this hypothesis, we treated late 1-cell stage NT embryos with three H3K27me3 inhibitors, which are EED226 (a PRC2 inhibitor that binds to the K27m3-pocket on EED and inhibits H3K27me3 methylation), UNC1999 (an EZH1/2 inhibitor), and Valemetostat (another EZH1/2 inhibitor), following by producing Smart-seq2 when the embryos reached the late 2-cell stage. To confirm the depletion of H3K27me3 by these inhibitors, we firstly conducted immunostaining of H3K27me3 in NT 2-C embryos with and without inhibitor treatment (Fig. S8B and S8C). The results showed that with the addition of EED226 (EED panel in Fig. S8B and S8C) and Valemetostat (Val panel in Fig. S8B and S8C), the H3K27me3 signal intensity in NT 2-C decreased significantly, particularly after the addition of Valemetostat. Then, we compared the expression levels of NT 2-C ueH3K27me3 genes and all genes (as control) in NT 2-C, NF 2-C, and NT 2-C by treating with 3 inhibitors, which are NT 2-C EED (2-cell stage NT embryos treated with EED226), NT 2-C UNC (2-cell stage NT embryos treated with UNC1999), and NT 2-C Val (2-cell stage NT embryos treated with Valemetostat). The results showed that the addition of all three inhibitors significantly elevated the expression levels of ueH3K27me3 genes compared to NT 2-C embryos without H3K27me3 inhibitor treatment (Fig. S8D). However, this phenomenon was not occurred when we compared expression levels of all genes among the five groups above (Fig. S8E). Although global H3K27me3 signal intensity did not show a significant change following the addition of UNC1999 (UNC panel in Fig. S8B and S8C), Fig. S8E indicated a clear recovery in the expression levels of ueH3K27me3 genes after UNC1999 treatment. The addition of H3K27me3 inhibitors effectively elevated the expression levels of genes specifically marked by high H3K27me3 signals in NT 2-C embryos. This suggests that abnormalities in H3K27me3 in NT embryos may play a role in the aberrant downregulation of ueH3K27me3 genes. Notably, these genes accounted for a large portion of all downregulated genes in NT embryos. Gene Ontology analysis unraveled that these ueH3K27me3 genes are enriched in functions related to embryo development, such as blastocyst formation, blastocyst hatching, and *in utero* embryonic development (Fig. S8F). Notably, ueH3K27me3 genes in the 2-cell stage exhibited a substantial overlap with ZGA genes (260 out of 415, 62.65%) (Fig. 4E), prompting the hypothesis that over-represented H3K27me3 may affect ZGA in NT embryos. Noteworthy examples of ZGA genes include *Kdm5a* and *Smarcc1* (Fig. 4F), which display higher H3K27me3 signals at promoter regions and lower expression levels in NT 2-C compared with NF 2-C. *Kdm5a* functions as an H3K4me3-specific demethylase (Li et al., 2014), while *Smarcc1* encodes one of the core components of the SWI/SNF complex associated with chromatin structure reorganization (Panamarova et al., 2016). This disruption of H3K27me3 may consequently perturb chromatin structure and potentially contribute to the excessive establishment of H3K4me3 in 2-cell stage NT embryos (Fig. S8G). Further exploration is warranted to elucidate these intricate molecular dynamics. Collectively, we observed significant H3K27me3 reprogramming defects during NT embryo development, especially in the 2-cell stage embryos. These aberrations in H3K27me3 reprogramming events might exert an influence on NT embryo development by disrupting the expression of ZGA genes, consequently leading to the dysregulation of expression patterns in downstream genes associated with embryo development.

### Bivalent genes are abundantly enriched in NT 2-cell embryos

**Figure 5.**
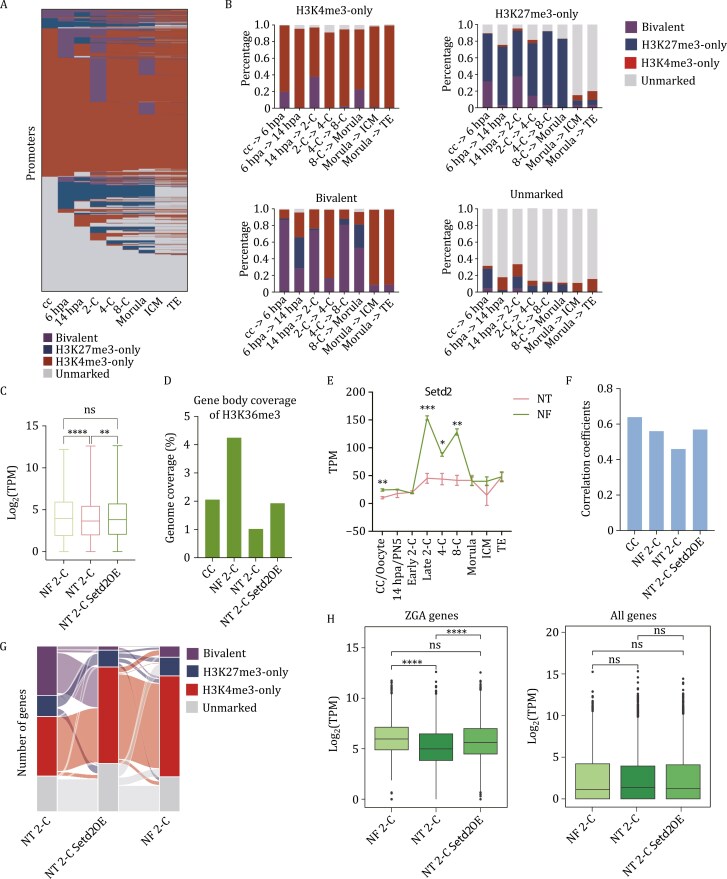
**Overexpression of *Setd2* restores disorganized epigenetic marks and ZGA gene expression in NT embryos.** (A) Heatmap illustrating the dynamics of bivalent genes during mouse NT embryo development. Each row corresponds to one RefSeq gene. (B) Bar chart depicting the tendencies of the H3K4me3-only promoters, H3K27me3-only promoters, bivalent promoters, and unmarked promoters in the subsequent stage during NT embryo development. Each bar represents the type and percentage of genes in the next stage. (C) Box plot displaying expression levels of NT 2-C bivalent genes in NF 2-C, NT 2-C, and NT 2-C *Setd2*OE samples. The expression levels were evaluated using an averaged log_2_(TPM). Significance was analyzed by using Student’s *t*-test (ns: not significant, **P < 0.01, ****P < 0.0001). (D) Bar chart displays the proportion of the genome covered by H3K36me3 in gene body regions in CC, NF 2-C, NT 2-C, and NT 2-C *Setd2*OE embryos. (E) Line chart presenting the expression levels (TPM) of *Setd2* in NT and NF pre-implantation embryos, CCs, and oocytes. Data represented as mean ± SD. (F) Bar chart shows the correlation coefficients between gene expression and H3K36me3 signal values at gene body regions in CC, NF 2-C, NT 2-C and NT 2-C *Setd2*OE embryo samples. (G) Alluvial plot illustrating the dynamics of H3K4me3-only, H3K27me3-only, bivalent and unmarked genes among NT 2-C, NF 2-C and 2-cell stage NT embryos with *Setd2* overexpression (NT 2-C *Setd2*OE). (H) Bar charts showing expression levels of ZGA genes (left panel) and all genes (right panel) in NF 2-C, NT 2-C, and NT 2-C *Setd2*OE samples. The expression levels were evaluated using an averaged log_2_(TPM). Significance was analyzed by using Student’s *t*-test (ns: not significant, ****P < 0.0001).

Bivalent genes, characterized by the co-occupancy of the H3K4me3 and H3K27me3 marks, were initially identified in embryonic stem cells (ESCs) and proposed to prime development regulator genes for subsequent transcriptional activation during differentiation (Azuara et al., 2006; Bernstein et al., 2006). Given the accumulation of both H3K4me3 and H3K27me3 marks at promoters in 2-cell stage NT embryos, we investigated the presence of bivalent domains at promoter regions. Indeed, a large portion of bivalent genes were observed in 2-cell stage NT embryos (Fig. 5A; Table S4), while bivalent genes were barely found in NF embryos (Liu et al., 2016b). Similar to NF embryos, the dynamics of bivalent domains were primarily attributed to the deposition or elimination of H3K27me3 signals (Fig. 5B). Considering the transcriptional repression role of H3K27me3, it implies that these bivalent genes may display lower expression levels compared to NF embryos at the same developmental stage. We compared the expression levels of NT 2-C bivalent genes between NT 2-C and NF 2-C. Indeed, these genes are significantly downregulated in NT 2-C compared to NF 2-C (Fig. 5C), indicating a disrupted gene expression pattern related to aberrant bivalent marks at promoters in NT embryos.

### Overexpression of *Setd2* can prevent the over-deposition of H3K4me3 and H3K27me3

Previous studies have underscored the antagonistic relationship between H3K36me3 and H3K4me3/H3K27me3 (Schmitges et al., 2011; Xie et al., 2011; Xu et al., 2019). We next sought to explore whether the over-enrichment of both H3K4me3 and H3K27me3 is correlated with H3K36me3. To delve deeper into this, we examined H3K36me3 enrichment in NT and NF 2-cell stage embryos as well as CCs using ULI-NChIP (Fig. S9A; Tables S1 and S2). Remarkably, NT 2-cell embryos exhibited a significant deficiency in gene body regions compared to NF 2-cell embryos (Fig. 5D). The deficiency could be attributed to the inherently low levels of H3K36me3 in CCs (Fig. 5D) and the apparently lower level of *Setd2* expression in NT 2-cell embryos compared to NF 2-cell embryos (which encodes the primary methyltransferase of H3K36me3 *in vivo* (Hu et al., 2010)) (Figs. 5E and S9B). Indeed, H3K36me3 was found to be mutually exclusive with both H3K4me3 and H3K27me3 in 2-cell stage NT embryos (Fig. S9C), and H3K36me3 signal at gene body regions is positively correlated with gene expression (Fig. 5F). This suggests that the impaired H3K36me3 deposition might contribute to the over-establishment of both H3K4me3 and H3K27me3, eventually affecting gene expression pattern in NT embryos.

Subsequently, *Setd2* mRNA was injected into reconstructed embryos, and 2-cell stage NT embryos were collected for ULI-NChIP of H3K4me3, H3K27me3, H3K36me3, along with Smart-seq2 for transcriptome profiling (Fig. S9D; Tables S1 and S2). *Setd2* was evidently overexpressed (Fig. S9E), and replicates of the above data correlated well (Fig. S9F). Following *Setd2* overexpression, there was a modest increase in the genomic coverage of H3K36me3 at gene body regions (Fig. 5D). Expectedly, with *Setd2* overexpression, the distribution of H3K4me3 and H3K27me3 modifications in the gene promoter regions of NT embryos more closely resembled that of 2-cell stage NF embryos (Figs. 5G and S10A–D). Specifically, the pattern of genes marked by H3K27me3 closely mirrored that of NF 2-cell stage embryos with *Setd2* overexpression (Fig. S10B and S10D), consequently reducing the number of over-represented bivalent genes (Fig. 5G). Additionally, the expression levels of NT 2-C bivalent genes were significantly elevated to the levels observed in NF 2-C embryos (Fig. 5C). This suggests that the injection of *Setd2* mRNA successfully mitigated the over-representation of both H3K4me3 and H3K27me3.

Moreover, principal component analysis (PCA) revealed the reinstatement of gene expression patterns in 2-cell stage NT embryos upon *Setd2* overexpression (Fig. S10E). Notably, the expression profiles of ZGA genes were significantly rejuvenated (Fig. 5H), indicating the pivotal regulatory role of *Setd2* in facilitating ZGA events. Hence, the overexpression of *Setd2* resulted in the rescue of multiple epigenetic markers, encompassing H3K36me3, H3K4me3, and H3K27me3, subsequently restoring ZGA-related gene expression patterns in 2-cell stage NT embryos.

### Overexpression of *Setd2* eliminates H3K27me3 at bivalent gene bodies to improve SCNT efficiency

**Figure 6.**
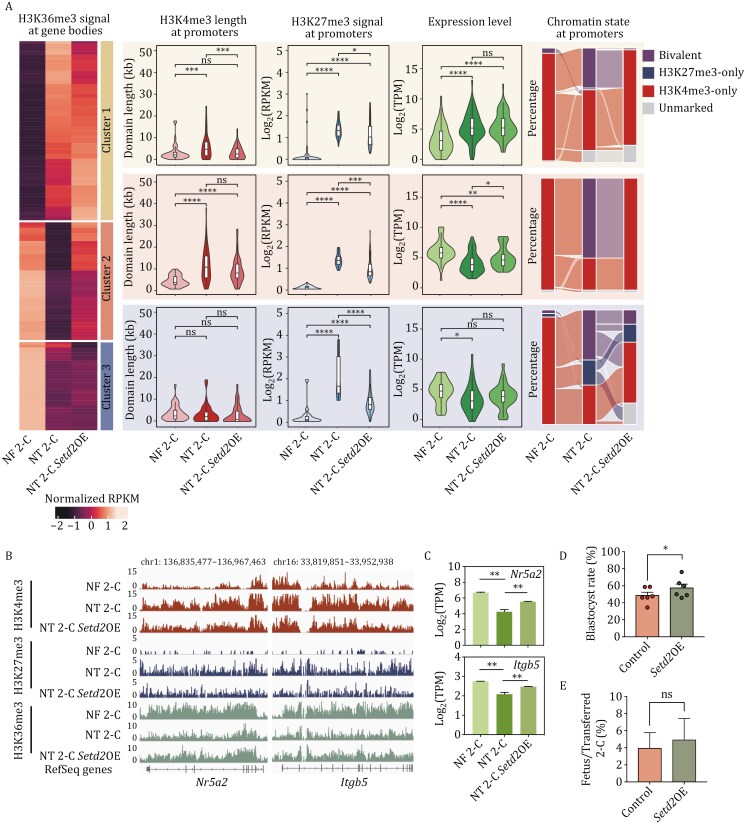
**Overexpression of *Setd2* excludes H3K27me3 by depositing H3K36me3 at bivalent gene bodies.** (A) Heatmap (the H3K36me3 panel) displaying clustering results based on the enrichment of H3K36me3 at gene body regions in 2-cell stage NF embryos (NF 2-C), 2-cell stage NT embryos (NT 2-C) and 2-cell stage NT embryos with *Setd2* overexpression (NT 2-C *Setd2*OE) samples. Violin plots demonstrating H3K4me3 domain length (the H3K4me3 domain length panel), H3K27me3 enrichment (the H3K27me3 panel) at promoter regions, and expression levels of genes (the RNA panel) in each cluster. Alluvial plots (the chromatin state panel) illustrating the dynamics of H3K4me3-only, H3K27me3-only, bivalent, and unmarked genes among NT 2-C, NF 2-C, and NT 2-C *Setd2*OE samples. Significance analyzed using Student’s *t-*test (ns: not significant, *P < 0.05, **P < 0.01, ***P < 0.001, ****P < 0.0001). (B) Snapshots showing H3K4me3, H3K27me3, and H3K36me3 signal in NF 2-C, NT 2-C and NT 2-C *Setd2*OE for *Nr5a2* and *Itgb5*. (C) Bar chart showing gene expression levels of *Nr5a2* and *Itgb5* in NF 2-C, NT 2-C, and NT 2-C *Setd2*OE. Data represented as mean ± SD. (D) Bar chart illustrating the percentage of embryos reaching the blastocyst stage for NT (control) and NT *Setd2*OE (*Setd2*OE) samples. Data represented as mean ± SD (*n* = 6). Significance analyzed using Student’s *t*-test (*P < 0.05). (E) Bar chart displaying birth rates of NT embryos (control) and *Setd2*-overexpressed NT embryos (*Setd2*OE). Birth rates are calculated as the number of fetuses divided by the number of 2 cells transferred to recipients. Data are represented as mean ± SD (*n* ≥ 3). Significance was analyzed by using Student’s *t*-test (ns: not significant).

Next, we investigated whether the rescue of H3K36me3 functionally contributes to the restoration of gene expression patterns. Pairwise comparisons of H3K36me3 signals at gene body regions in 2-cell stage NF embryos, 2-cell stage NT embryos and 2-cell stage NT embryos with *Setd2* overexpression categorized genes into three clusters (Fig. 6A). For genes in cluster 1, H3K36me3 signals at their gene bodies were enriched in 2-cell stage NT embryos compared to 2-cell stage NF embryos and remained at high levels upon *Setd2* overexpression. Conversely, for genes in cluster 3, H3K36me3 signals at their gene bodies were deficient in 2-cell stage NT embryos compared to 2-cell stage NF embryos and remained at low levels upon *Setd2* overexpression. Given the established knowledge that H3K36me3 signals at gene body regions correlate with gene expression levels (Xu et al., 2019), despite the shortening of H3K4me3 domain length or weakening of H3K27me3 signals at promoter regions by *Setd2* overexpression, the aberrantly high expression levels of genes in cluster 1 and low expression levels of genes in cluster 3 in 2-cell stage NT embryos has not changed significantly. This may be attributed to the aberrant H3K36me3 signals at their gene body regions (Fig. 6A). Genes in cluster 2, primarily composed of bivalent genes, exhibited rescued H3K36me3 signals at gene body regions upon *Setd2* overexpression, leading to a significant restoration of their expression levels (Fig. 6A). This suggests that overexpression of *Setd2* can help remove H3K27me3 from bivalent genes, while the restoration of gene expression levels requires the establishment of H3K36me3 modifications on the gene body. For instance, *Nr5a2,* an essential pioneer factor regulating ZGA and pluripotency (Gassler et al., 2022; Li et al., 2024), and *Itgb5*, an early TE gene highly expressed in the early stages of mouse embryos (Li et al., 2024), displayed underexpression and deficient H3K36me3 signals at gene body regions, along with high H3K27me3 signals at promoter regions in 2-cell stage NT embryos. However, overexpression of *Setd2* successfully rescued these epigenetic marks, leading to the subsequent elevation of gene expression levels (Fig. 6B and 6C). Similarly, the restoration of ZGA gene expression levels was associated with the reinstatement of H3K36me3 at gene body regions to exclude H3K27me3 at promoter regions upon *Setd2* overexpression (Figs. 5H and S10F). Furthermore, despite incomplete restoration of H3K36me3 in some gene bodies (Fig. 6A, cluster 3), H3K27me3 was still removed from promoters of these genes, indicating additional underlying mechanisms for H3K27me3 restoration. Nevertheless, our findings indicate that the simultaneous removal of overrepresented H3K27me3 at bivalent promoters and the recovery of H3K36me3 at these gene bodies are crucial for rescuing gene expression patterns (Fig. 6A). Lastly, the observed enhancement in cloning efficiency following the overexpression of *Setd2* in NT embryos (Fig. 6D and 6E) underscores the potential of *Setd2* overexpression to improve cloning efficiency.

## Discussion

**Figure 7.**
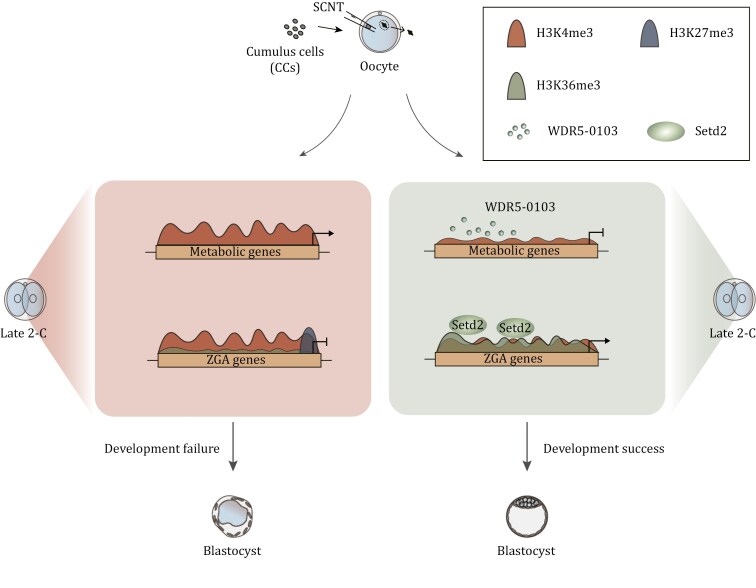
Model illustrating epigenetic reprogramming during NT embryogenesis by WDR5-0103 addition and ***Setd2*** overexpression. The figure was created with Adobe Illustrator.

Despite the tremendous potential applications of SCNT technology, cloning efficiency remains low in most species, and the mechanisms underlying epigenetic reprogramming following SCNT remain largely elusive (Matoba and Zhang, 2018; Zhang et al., 2021). In this study, we observed an unexpected rapid establishment of both H3K4me3 and H3K27me3 genome-wide in the early stages of SCNT pre-implantation embryos. This led to prolonged H3K4me3 domains and stronger H3K27me3 signals at promoter regions, thereby disrupting the regulation of numerous genes associated with metabolism and embryo development, respectively. Comparative analysis with naturally fertilized embryos revealed major reprogramming defects of H3K4me3 existing at the 2-cell stage and earlier, while H3K27me3 reprogramming was impaired throughout early embryogenesis in NT embryos. These findings suggest that the over-representation of both active mark (H3K4me3) and repressive mark (H3K27me3) dramatically disrupts gene expression patterns in NT embryos and may act as reprogramming barriers that impede SCNT-mediated reprogramming (Fig. 7). Interestingly, we found that H3K4me3 is abnormally over-established in 2-cell stage NT embryos, not only at promoter regions of obH3K4me3 genes but also on some downegulated genes. These genes are highly expressed in NF embryos with broad H3K4me3 domains. A possible explanation for this discrepancy is that the further extension of broad H3K4me3 signals on these genes does not lead to a further increase in gene expression levels. On the other hand, the addition of certain repressive modifications, such as H3K27me3 and H3K9me3, leads to decreased expression. However, gene expression regulation is a complex process, and other epigenetic modifications or regulatory factors may also be involved, which requires further investigation.

The term “bivalency” was coined in 2006 to describe the co-occurrence of H3K4me3 and H3K27me3 at promoters of developmental transcription factors that are lowly expressed in mouse ESCs (Bernstein et al., 2006). Subsequent work expanded the concept of bivalent promoter genome-wide in both mouse and human ESCs (Mikkelsen et al., 2007; Pan et al., 2007; Zhao et al., 2007), and then to pluripotent cells and various other cell types (Lesch et al., 2013; Liu et al., 2016b; Roh et al., 2006; Rugg-Gunn et al., 2010; Xiang et al., 2020). Bivalency is thought to poise key developmental regulatory genes for future activation or repression, depending on the cell fate trajectory (Azuara et al., 2006; Bernstein et al., 2006). A bivalent chromatin signature evident in gametes is lost after fertilization, owing to loss of H3K27me3 from conventional Polycomb targets, promoters of crucial development and differentiated genes, and begins to emerge in the ICM, with a clearly ESC-like pattern evident by the post-implantation epiblast stage (Xiang et al., 2020; Zheng et al., 2016). Surprisingly, we observed numerous genes with bivalency at their promoters in 2-cell stage NT embryos, an important stage with the onset of major ZGA. Indeed, a substantial proportion of ZGA-related genes are marked by both H3K4me3 and H3K27me3 marks at their promoter regions, which may lead to the compromised expression levels of these genes. We also found the dynamics of bivalency during pre-implantation NT embryo development are mainly due to the deposition and elimination of H3K27me3. Considering the role of PRC2 complex in depositing H3K27me3 in pre-implantation embryos (Liu et al., 2016b) and the low expression levels of *Eed*, *Suz12*, and *Ezh2* in CCs, the accumulation of bivalent genes in 2-cell stage NT embryos may be due to the pre-transcription of these genes in oocyte cytoplasm.

The interactions between epigenetic modifications and their regulatory effects on gene expression present an interesting scientific question. Previous studies have underscored the pivotal role of *Setd2* as a central player in regulating the epigenome across various contexts (Mukai et al., 2015; Xu et al., 2019). Mice deficient in SETD2 typically perish around embryonic day 10.5–11.5 (Hu et al., 2010). Maternal depletion of SETD2 results in oocyte maturation defects and subsequent one-cell arrest after fertilization (Xu et al., 2019). Importantly, *Setd2* deficiency leads to the invasion of H3K4me3 and H3K27me3 into former H3K36me3 territories (Xu et al., 2019). Given the antagonistic relationship between H3K36me3 and H3K4me3/H3K27me3, our findings suggest that the excessive accumulation of H3K4me3 and H3K27me3 in 2-cell stage NT embryos can be attributed to *Setd2* deficiency and the loss of H3K36me3. Notably, the overexpression of *Setd2* in NT embryos effectively rectified the aberrant patterns of H3K36me3, H3K4me3, and H3K27me3, thereby enhancing cloning efficiency by restoring gene expression patterns in NT embryos. The underlying molecular mechanisms may involve the recruitment of KDM5B and the exclusion of PRC2 complex by H3K36me3 (Xie et al., 2011; Yuan et al., 2011). Previous studies have shown that PRC2 complexes are inhibited by H3K36me3 in both mammals and flies (Schmitges et al., 2011). Another study found that in HeLa cells, H3K27me3 rarely coexists with H3K36me3 on the same histone H3 polypeptide and that preexisting H3K36 methylation effectively inhibits PRC2-mediated H3K27 methylation *in vitro* (Yuan et al., 2011). Considering that the time point of *Setd2* injection is before the 2-cell stage, when H3K27me3 has not yet accumulated, the overexpression of *Setd2* may lead to the restored H3K36me3 directly interfering with the function of PRC2, thereby preventing the establishment of H3K27me3 in NT 2-C embryos. However, the overexpression of *Setd2* in NT embryos resulted in a lesser correction of H3K4me3 compared to the correction of H3K27me3, suggesting the involvement of other mechanisms in the excessive establishment of H3K4me3. Furthermore, we observed that bivalent promoters in 2-cell stage NT embryos are more likely to be targeted by exogenous *Setd2*, leading to the accumulation of H3K36me3 at gene body regions and exclusion of H3K27me3 from their promoters, ultimately restoring expression patterns. It is worth noting that numerous ZGA genes confirm this pattern, including two important pioneer genes associated with embryo development, namely *Nr5a2* and *Itgb5*. Our findings underscore the central role of *Setd2* in regulating the epigenetic reprogramming of ZGA during NT embryo development.

H3K9me3 is another repressive histone mark that plays a crucial role in embryo development and often decorates constitutive heterochromatin. In a previous study, Shogo Matoba et al. first identified donor-inherited H3K9me3 as a major barrier to SCNT reprogramming, leading to a large proportion of reprogramming resistant regions (RRRs) in 2-cell stage SCNT embryos (Matoba et al., 2014). Recently, we have pushed this barrier forward to minor ZGA (Xu et al., 2023). Another previous study of ours indicated that donor-inherited H3K9me3 impedes the removal of topologically associated domains (TADs) during SCNT embryo development, while overexpression of *Kdm4b*, an H3K9me3 demethylase, partially ameliorate abnormal 3D chromatin structures (Chen et al., 2020). Whether the abnormal 3D chromatin structure caused by aberrant H3K9me3 reprogramming further affects other histone marks remain unclear. Additionally, in oocytes, loss of KDM4B alters the global H3K9me3 landscape at sites occupied by broad domains of H3K4me3 (bdH3K4me3), providing a prime example of crosstalk between H3K9me3 and H3K4me3 (Sankar et al., 2020). It would be interesting to explore the crosstalk between H3K9me3 and H3K4me3 in the context of embryos, and whether the aberrant H3K9me3 inherited from donor cells is the initial obstacle in SCNT reprogramming that subsequently disrupts the normal hierarchy of other histone marks, such. as H3K4me3, H3K27me3, and H3K36me3. Further studies should investigate the regulatory networks and crosstalk across numerous epigenetic marks.

Taken together, our study uncovered the incomplete reprogramming of H3K4me3 and H3K27me3, which disrupts gene expression patterns and developmental potential in cloned embryos. Through the overexpression of *Setd2*, we effectively restored expression levels of genes crucial for ZGA and embryo development. This restoration was achieved by enhancing H3K36me3 at gene body regions and excluding H3K27me3 from bivalent promoters, ultimately improving cloning efficiency. Our findings illuminate the defects and molecular principles underlying the establishment of the epigenome during SCNT-mediated embryogenesis and offer insights into the interplay among H3K36me3, H3K4me3, and H3K27me3. Further studies should aim to provide a comprehensive understanding of the crosstalk among other epigenetic configurations and validate the roles of hierarchical epigenetic rearrangements during SCNT-mediated reprogramming.

## Methods

### Mice and donor cells

Specific-pathogen-free (SPF) mice were housed in the animal facility at Tongji University, Shanghai, China. All animal maintenance and experimental procedures were performed according to Tongji University Guide for the use of laboratory animals. Mouse were housed under 12-h light/dark cycle under pathogen-free conditions at (22 ± 2)°C and fed with free access to standard mouse chow and tap water.

B6D2F1 (C57BL/6 female × DBA2 male) female mice at 8–10-week-old were used as oocyte recipients. Cumulus cells (female) were collected from B6D2F1 background mice. 9–15-week-old females from the ICR strain were used as recipients for embryo transplantation.

Both cumulus cells and oocytes were prepared by super-ovulating 8–10-week-old B6D2F1 female mice. Super-ovulation was induced by the sequential injection with 7 IU each of pregnant mare serum gonadotropin (PMSG) and 5 IU each of human chorionic gonadotropin (hCG) at intervals of 48 h. Cumulus-oocyte complexes (COCs) were collected from oviducts 14 h after hCG injection and treated with bovine testicular hyaluronidase to obtain dissociated cumulus cells and oocytes, respectively.

### Somatic cell nuclear transfer and embryo culture

MII oocytes were collected from super-ovulated adult B6D2F1 females and cultured in Chatot-Ziomek-Bavister medium (CZB) at 37°C and 5% CO_2_ until use. Cumulus cells were collected in HEPES-CZB (HCZB) and stored at 4°C until nuclear transfer manipulation. The oocytes were enucleated in HCZB containing 5 μg/mL cytochalasin B (CB) (Sigma) by a Piezo-driven pipette at 37°C under an Olympus inverted microscope (Tokyo, Japan). Then, the donor cumulus cells were injected into enucleated oocytes. Reconstructed embryos were cultured for 1 h in CZB at 37°C and 5% CO_2_ and then activated by incubation in Ca^2+^-free CZB containing 1 mmol/L SrCl_2_ and 5 μg/mL CB for 5–6 h. After activation, the reconstructed embryos were washed 5–7 times and cultured in G1 medium at 37°C and 5% CO_2_.

### *In vitro* transcription of mRNA and direct injection

Mouse *Setd2* mRNA were cloned into T7-driven vectors and mRNAs were synthetized *in vitro* by using mMESSAGE mMACHINE T7 Ultra Kit (Life Technologies, Grand Island, NY, USA) following the manufacturer’s instructions. The final concentration of mRNA was diluted to 500 ng/μL before injection. Enucleated oocytes were injected with ~10 pL of mRNA using a Piezo-driven micromanipulator.

### WDR5-0103 addition in cloned embryos

Final concentration of 20 mmol/L WDR5-0103 (Selleck Chemicals, S2184) was added into the culture medium immediately after activation and removed when the embryos reached to late 2-cell stage.

### H3K27me3 inhibitor addition in cloned embryos

Final concentration of 10 mmol/L EED226 (Selleck Chemicals, S8496), UNC1999 (MedChem Express, HY-15646) and Valemetostat (tosylate, MedChem Express, HY-109108A) were added into the culture medium at 14 h after activation until the embryos reached to late 2-cell stage, respectively.

### Embryo transfer

The *Setd2*-injected, WDR5-0103-added, and control NT embryos at 2-cell stage were transferred into the oviduct of pseudo-pregnant female mice, respectively. Caesarean section was carried out at day 19.5.

### Immunofluorescence analysis

Cumulus cells and embryos at each stage—6 h and 14 h post-activation, late-2-cell (30–32 h post-activation), 4-cell (38–42 h post-activation), 8-cell (56 h post-activation), morula and day 4 blastocyst—were fixed with 4% paraformaldehyde in PBS for 15 min at room temperature (RT). Then, the fixed samples were permeabilized with 0.2% Triton X-100 for 15 min at RT. And then, the samples were blocked with 1% bovine serum albumin (Sigma) in phosphate-buffered saline (BSA-PBS) solution for 1 h and then incubated with the primary antibodies against H3K4me3 (Cell signaling, #9751) and H3K27me3 (Diagenode, C15410195) overnight at 4°C. After washing three times with PBS, the samples were incubated with the appropriate secondary antibodies at RT for 2 h. After washing two times with PBS, the samples were stained with DAPI (Millipore) for 20 min at RT. After washing twice as above, the samples were observed for fluorescence under a laser-scanning confocal microscope (LSM880, Zeiss). For immunostaining for OCT4, CDX2, and SETD2, anti-Oct4 antibody (Abcam ab181557), anti-CDX2 antibody (BioGenex MU392A-5UC), and SETD2 polyclonal antibody (ABclonal, A3194) were used in this study.

### ULI-NChIP-seq

For ULI-NChIP-seq, 500 cells were used per reaction, and two replicates were performed for each stage. All isolated cells were washed three times in 0.5% BSA-PBS to avoid potential contamination. The ULI-NChIP procedure was performed as previously described (Brind’Amour et al., 2015). One microgram of either histone H3K4me3 antibody (Cell signaling, #9751), H3K27me3 antibody (Diagenode, C15410195), or H3K36me3 antibody (Abcam, AB9050) was used for each immunoprecipitation reaction.

The sequencing libraries were generated using the KAPA Hyper Prep Kit (KAPA, KK8504, Switzerland) for the Illumina platform, following the manufacturer’s instructions. Paired-end sequencing with a 150 bp read length was performed on a NovaSeq (Illumina) platform.

### SMART-seq2 RNA-seq

According to previous methods, approximately 10–20 cells were used to construct each library (Picelli et al., 2014). The prepared cells were transferred to the lysis buffer by mouth pipetting. Reverse transcription was performed directly using oligo (dT) primers in lysates containing compete cytoplasm. Second-strand cDNA was synthesized depending on the poly (A) tail attached to the 3’ end of the first-strand cDNA or via template switching with a TSO. Fragment cDNA was prepared for sequencing library construction using a Covaris sonicator (Covaris S220, Wobum, MA, USA).

The sequencing libraries were generated using the KAPA Hyper Prep Kit (KAPA, KK8504, Switzerland) for the Illumina platform, following the manufacturer’s instructions. Paired-end sequencing with a 150 bp read length was performed on a NovaSeq (Illumina) platform at.

### ChIP-seq and RNA-seq data processing

ChIP-seq reads were first subjected to Trim_galore (version 0.6.4) for adaptor trimming as well as quality control with parameters --paired -j 8. The trimmed paired-end reads were aligned to the mouse mm10 reference genome using the bwa (version 0.7.17) (Li, 2013) mem command. PCR duplicates were removed by MarkDuplicates of Picard Tools (version 2.21.1) and only high-quality mapped reads (MAPQ > 30) were retained for downstream analyses. Correlation of the normalized signal intensity between biological replicates were calculated on all RefSeq gene promoters, which are defined as ± 2 kb around TSSs. Highly correlated biological replicates were pooled together for each stage and performed for downstream analyses. ChIP-seq peaks were identified by MACS2 (version 2.2.4) (Zhang et al., 2008) callpeak command using the parameters --broad --nolambda --nomodel -g mm. H3K36me3 domains were than merged from peaks with a distance < 5 kb using bedtools (version 2.28.0) (Quinlan and Hall, 2010) merge command.

RNA-seq reads were first subjected to Trim_galore (version 0.6.4) for adaptor trimming as well as quality control with parameters --paired -j 8. The trimmed paired-end reads were aligned to the mouse mm10 reference genome using HISAT2 (version 4.8.2) (Kim et al., 2019). Multiple mapped reads were filtered out. Uniquely mapped reads were then used to calculate fragments per kilobase per million (FPKM) to represent gene expression levels for each sample using StringTie (version 2.1.1) (Pertea et al., 2016). For downstream data analysis, FPKM values were averaged for each gene between replicates.

Differentially expressed genes (DEGs) were defined with stringent cutoffs of *P.adj *< 0.01 and fold change > 5 using the R package DESeq2 (Love et al., 2014).

### ChromHMM analysis and alluvial plotting

Chromatin states were identified and characterized using ChromHMM (version 1.20) (Ernst and Kellis, 2012). The alignment files of H3K4me3 and H3K27me3 modifications of all samples were binned into 200 bp bins using the BinarizeBam command, with the input alignment file as control. A stringent threshold (fold enrichment greater than four using -f 4 option for H3K4me3 samples and -f 2 option for H3K27me3 samples) to remove the low-enrichment domains. Next, we trained the model with 4 emission states using 200 bp resolution and default parameters using the LearnModel command. Finally, at each stage, the whole genome was classified into four states: H3K4me3-only (without H3K27me3) regions, H3K27me3-only (without H3K4me3) regions, bivalent (with both H3K4me3 and H3K27me3) regions and unmarked (with neither H3K4me3 nor H3K27me3) regions.

The segmentation file of each stage was further binned to 200 bp intervals to calculate the number of transitions between chromatin states during development or differentiation. Alluvial diagrams of developmental and differentiation lineages were plotted using the alluvial function in *R* to show the transitions. The total regions of each chromatin state were counted once the 200 bp intervals had been marked by the specific states during that lineage, and the percentages of specific intervals in each stage were plotted to show the global trend of that specific chromatin state. The alluvial diagrams showed the percentage changes of chromatin states during each transition; the lines from the present stage to next stage cannot be traced, as they represent different genomic locations.

### Classification of genes and visualization

*RefSeq* genes were classified based on their promoters overlapping with ChromHMM segments in each stage. Promoters were defined as ± 2 kb around transcription start site (TSS). H3K4me3 marked genes were defined once their promoter regions have > 200 bp overlaps with H3K4me3-only regions or bivalent regions; H3K27me3 marked genes were defined once their promoter regions have > 200 bp overlaps with H3K27me3-only regions or bivalent regions; bivalent marked genes were defined once their promoter regions have > 200 bp overlaps with bivalent regions or they overlapped with both H3K4me3-only and H3K27me3-only regions; the remained genes were defined as control genes.

To visualize genes based on their chromatin states, the gene matrices of the developmental stages were transformed into 0/1 matrices based on their chromatin status, with 1 represents H3K4me3-marked genes and 0 represents non-marked genes, with the same criteria for the H3K27me matrix. All genes were then ranked based on their score to generate heat maps. A similar quaternary transition method was used for bivalent and H3K4me3 breadth visualization.

### Classification of H3K4me3 marked genes

H3K4me3 marked genes were classified into four groups bases on the segment lengths at their promoter regions. Genes overlapped with segments > 5,000 bp were defined as broad H3K4me domain genes; those overlapped with segments ≤ 5,000 bp and > 1000 bp were defined as medium H3K4me3 domain genes; those overlapped with segments ≤ 1000 bp and > 200 bp were defined as narrow H3K4me3 domain genes; and those overlapped with segments ≤ 200 bp were defined as control H3K4me3 domain genes.

### Mutually exclusive index

The global mutually exclusive index was calculated as the random coincidence of an H3K4me3 domain (or H3K27me3 domain) and an H3K36me3 domain versus real coincidence, *m*_*s*_ = *B*_*r*_*/B*_*v*_. Where


$$\begin{array}{l} B_{r}=\frac{nH3K4me3\left(or\;H3K27me3\right)\;\times \;nH3K36me3}{ntotal} \end{array}$$

$$\begin{array}{l} B_{v}=n_{bi-domain} \end{array}$$

in which *n*_*H3K4me3*_ (or *n*_*H3K27me3*_) stands for the number of H3K4me3-marked (or H3K27me3-marked) domains in 2-cell stage NT embryos, *n*_*H3K36me3*_ stands for the number of H3K36me3-marked domains, and *n*_*bi-domain*_ stands for the domains simultaneously marked by H3K4me3 (or H3K27me3) and H3K36me3.

### Clustering analyses

*K*-means clustering of gene expression and histone modification enrichment was conducted with Cluster (version 2.1.0). The hierarchical clustering analysis was performed based on Spearman’s Rank Correlation.

### Gene ontology analysis

Gene ontology (GO) analysis was performed according to the Database for Annotation, Visualization and Integrated Discovery (DAVID) tools (Sherman et al., 2022). GO terms for each functional cluster were summarized to a representative term, and *P* values were plotted to show the significance.

### Statistical analyses

Statistical analyses were implemented with *R* (version 3.6.3). Pearson’s *r* coefficient was calculated using the “cor” function with default parameters. Quantitative analyses were performed with Prism (version 7.0a) (GraphPad Software) with at least three independent experiments and presented as the mean ± SD. The *P* values between two groups of samples were calculated using a two-tailed unpaired Student’s *t*-test.

## Supplementary data

Supplementary data is available at *Protein & Cell* Journal online https://doi.org/10.1093/procel/pwaf010.

pwaf010_suppl_Supplementary_Materials

pwaf010_suppl_Supplementary_Tables_S1

pwaf010_suppl_Supplementary_Tables_S2

pwaf010_suppl_Supplementary_Tables_S3

pwaf010_suppl_Supplementary_Tables_S4

## Data Availability

The generated and analyzed datasets in the current study are available in the Gene Expression Omnibus (GEO) with accession number GSE262040. The accession numbers of public RNA-seq datasets of fertilized embryos and SCNT embryos are GSE71434 and GSE195762, respectively. The accession number of public H3K4me3 and H3K27me3 ChIP-seq datasets of fertilized embryos are GSE73952 and GSE97778.

## Code availability

Codes are available from the corresponding authors upon reasonable request.
