# Supplementary material for: Setd2 overexpression rescues bivalent gene expression during SCNT-mediated ZGA
Source: Protein Cell. 2025 Feb 13;16(6):439–57. doi: 10.1093/procel/pwaf010 (PMC12187520; doi:10.1093/procel/pwaf010)
Supplement: pwaf010_suppl_Supplementary_Materials [file pwaf010_suppl_supplementary_materials.pdf]

Supplementary materials

Figure S1. Ultra-low-input ChIP-seq in pre-implantation NT embryos.

(A) Schematic representation of pre-implantation NT embryos and donor cells (cumulus cells, CC) used in ULI-NChIP analysis.

(B) Correlation of H3K4me3 and H3K27me3 samples for SCNT embryos developmental stages and donor cells. Each point represents log2 RPKM of a RefSeq promoter for the replicates. Pearson's correlation coefficients of the two replicates were calculated using all RefSeq promoters.

(C, D) Genome browser view of H3K4me3 (C) and H3K27me3 (D) enrichment in SCNT pre-implantation embryos and CCs (with two replicates for each stage). The H3K4me3 and H3K27me3 enrichment is calculated as RPKM.

Figure S2. Features of chromatin states in pre-implantation NT and NF embryos.

(A, B) Alluvial plots (left panel) displaying the global dynamics of bivalent (A) and unmarked (B) regions during NT early embryo development. Each line represents a 200 bp bin defined on the ChromHMM categories, and the total regions are those classified as this state in at least one analyzed stage. The global dynamics (solid line) and chromosome X dynamics (dashed line) were plotted separately (right panel).

(C) Alluvial plots depict the global dynamics of H3K4me3-only (the first panel), H3K27me3-only (the second panel), bivalent (the third panel) and unmarked (the fourth panel) regions between morula and TE in NT embryos. Each line represents a 200 bp bin defined by ChromHMM categories, with total regions classified in this state in at least one analyzed stage (including CC, 6hpa, 14hpa, 2-C, 4-C, 8-C, morula, ICM and TE).

(D, E) Alluvial plots (left panel) displaying the global dynamics of bivalent (D) and unmarked (E) regions during NF early embryo development. Each line represents a 200 bp bin defined on the ChromHMM categories, and the total regions are those classified as this state in at least one analyzed stage. The global dynamics (solid line) and chromosome X dynamics (dashed line) were plotted separately (right panel).

(F, G) Line chart display averaged fluorescence signal intensity of H3K4me3 (F) and H3K27me3 (G) in each developmental stages of NF (green line) and NT (red line) embryos. The data are represented as mean ± SD (n>=3).

Figure S3. Discrepancies of genomic distribution of H3K4me3 and H3K27me3 between NT and NF embryos.

(A, B) Genome distribution of H3K4me3 (A) and H3K27me3 (B) in nuclear transfer (NT) and natural fertilized (NF) embryos, oocytes and CCs.

(C, D) Heatmap depicting fold change in expression levels of H3K4me3 (C) and H3K27me3 (D) writers and erasers between NT and NF embryos at the same developmental stage. The fold change was calculated using TPM values, where the expression level of a specific gene in NF embryos was divided by that in NT embryos.

Figure S4. Discrepancies of H3K4me3 domain width between NT and NF embryos.

(A) Dynamics of the H3K4me3 domain width on all RefSeq gene promoters throughout NF embryo development. Promoters are classified based on breadth of marked H3K4me3 domains: broad (dark red), medium (red), narrow (pink) and control (white).

(B) The percentage of broad H3K4me3 domains occupancy during NT early embryogenesis.

(C) The percentage of broad H3K4me3 domains occupancy during NF early embryogenesis.

(D) Tendencies of the four types of H3K4me3 domains in promoters in the next stage during NF embryogenesis. Each panel represents a specific type of H3K4me3 domain as marked above. Each bar represents the types and fractions of promoters in the next stage.

(E) Bar chart illustrating the comparison of averaged H3K4me3 domain width at RefSeq gene promoter regions between nuclear transfer (NT) and natural fertilized (NF) embryos. Significance was analyzed by using Student's *t* test (****$p < 0.0001$).

(F) Distribution of the H3K4me3 domain width at the promoter regions in NT embryos compared with the corresponding stage of NF embryos. Dashed lines represent the cut-off between medium and broad domains.

(G) Genome browser view displays H3K4me3 enrichment at the *Has2*, *Setd4* and *Zfp719* gene loci in NF 2-C and NT 2-C embryos. H3K4me3 enrichment is shown as scaled RPKM. With H3K4me3 domains presented by green bars.

Figure S5. Dysregulation of gene expression in NT 2-C embryos is correlated with aberrantly widened H3K4me3 domain.

(A) Box plot showing the averaged expression levels [log2 (TPM + 1)] of genes with broad, medium, narrow and control H3K4me3 domains at their promoter regions in each stage of NT embryos and donor cells, CCs.

(B) Gene Ontology (GO) analysis with dysregulated genes associated with aberrant H3K4me3 domains in each stage of NT embryos. obH3K4me3 genes: genes overexpressed with broadened H3K4me3 domains at their promoter regions; usH3K4me3 genes: genes under-expressed with shortened H3K4me3 domains at their promoter regions.

Figure S6. Correction of H3K4me3 domain width by addition of WDR5-0103 in NT embryos.

(A) Experimental schematic for the WDR5-0103 treatment. Two-cell stage NT embryos treated with 20 mM WDR5-0103 after activation were collected for H3K4me3 NChIP-seq and Smart-seq2.

(B) Scatter plot depicting the correlation of H3K4me3 signals between two replicates of 2-cell stage NT embryos with WDR5-0103 treatment (NT 2-C + WDR5-0103). Each point represents log2 RPKM of a RefSeq promoter for the replicates. Pearson's correlation coefficients of the two replicates were calculated using all RefSeq gene promoters.

(C, D) Correlation of Smart-seq2 samples for 2-cell stage (C) and 4-cell stage (D) NT embryos with WDR5-0103 treatment (NT 2-C + WDR5-0103; NT 4-C + WDR5-0103). Pearson's correlation coefficients of the two replicates were calculated using TPM of all RefSeq genes.

(E, F, G) Percentage of the whole genome (E), gene body regions (F) and intergenic regions (G)

marked by H3K4me3 in 2-cell stage nuclear transfer embryos (NT 2-C), 2-cell stage NT embryos with WDR5-0103 treatment (NT 2-C + WDR5-0103) and 2-cell stage of natural fertilized embryos (NF 2-C) samples.

Figure S7. Correction of gene expression and blastocyst quality by addition of WDR5-0103 in NT embryos.

(A) Screenshots showing H3K4me3 enrichment at the *Ephx2*, *Ndufc2* and *Spp1* gene loci in NF 2-C, NT 2-C and NT 2-C with WDR5-0103 addition (NT 2-C + WDR5-0103) embryos and expression level of *Ephx2*, *Ndufc2* and *Spp1* in NF 2-C, NT 2-C, NT 2-C + WDR5-0103, NF 4-C, NT 4-C and NT 4-C + WDR5-0103 embryos. H3K4me3 enrichment calculated as scaled H3K4me3 RPKM. Expression level calculated as FPKM. H3K4me3 domain presented as green bar.

(B) Box plot showing the percentage of OCT4+CDX2-/OCT4+ and OCT4-CDX2+/CDX2+ cells in control NT E4 blastocysts (Control) and NT E4 blastocysts with WDR5-0103 treatment at the 2-cell stage (WDR5-0103). Data are represented as mean ± SD (n >= 3). Statistical significance was assessed using Student's *t* test (ns: not significant; ****$p < 0.0001$).

Figure S8. Aberrant H3K27me3 domains impair gene expression in NT embryos.

(A) Scatterplots showing the comparison between gene expression and H3K27me3 enrichment at relative gene promoter at specific stages of NT versus NF embryos. Each point represents a RefSeq gene. Genes up-regulated in NT embryos compared to correlated NF embryos [log2FC > 2, *padj.* < 0.01, FC: Fold Change] with down-regulated H3K27me3 signal at promoter region [log2 (NT RPKM / NF RPKM) < -1] are plotted in red. Genes down-regulated in NT embryos compared to correlated NF embryos [log2FC < -2, *padj.* < 0.01] with up-regulated H3K27me3 signal at the promoter region [log2 (NT RPKM / NF RPKM) > 1] are plotted in blue.

(B) Immunostaining of H3K27me3 in 2-cell stage NT embryos with the addition of EED226 (EED panel), UNC1999 (UNC panel) and Valemetostat (Val panel). Two-cell stage NT embryos without addition of PRC2 inhibitor was used as control (Control panel). DAPI stains for DNA (blue) while H3K27me3 is visualized in green. A representative image from three independent experiments is presented. Scale bar: 50 µm. BF: Bright fild.

(C) Bar chart displays averaged fluorescence signal intensity of H3K27me3 in 2-cell stage NT embryos with the addition of EED226 (EED panel), UNC1999 (UNC panel) and Valemetostat (Val panel). Two-cell stage NT embryos without addition of PRC2 inhibitor was used as control (Control panel). The data are represented as mean ± SD (n>=3). Significance was analyzed by using Student's *t* test (ns: not significant; ****$p < 0.0001$).

(D, E) Bar chart showing expression levels of 2-C ueH3K27me3 genes (D) and all genes (E) in NT 2-C, NF 2-C, NT 2-C EED (2-cell stage NT embryos treated with EED226), NT 2-C UNC (2-cell stage NT embryos treated with UNC1999), and NT 2-C Val (2-cell stage NT embryos treated with Valemetostat). The expression levels were evaluated using an averaged log2TPM. Significance was analyzed by using Student's *t* test (ns: not significant, ****$p < 0.0001$).

(F) Gene Ontology (GO) analysis of ueH3K27me3 genes defined in Fig. S8A.

(G) Heatmap shows the H3K27me3 signal values at promoter regions (left) and domain length of

H3K4me3 over promoter regions (right) of ueH3K27me3 genes in NT 2-C and NF 2-C. H3K27me3 signal value is calculated as normalized H3K27me3 RPKM at promoter region.

Figure S9. Expression of *Setd2* is insufficient in 2-cell stage NT embryos compared to NF embryos.

(A) Scatter plot shows the correlation between H3K36me3 samples of CC (left panel), 2-cell stage natural fertilized embryos (NF 2-C) (medium panel) and 2-cell stage nuclear transfer embryos (NT) 2-C (right panel). Each point represents a genomic region of a 2-kb window. Pearson's correlation coefficients of the two replicates were calculated using log2 (RPKM) of H3K36me3 signal of all the 2-kb window regions.

(B) Bar chart showing the relative expression levels of *Setd2* in 2-cell NF and NT embryos. Quantitative PCR (qPCR) analysis was conducted using two pairs of primers (*Setd2*-1 and *Setd2*-2). Data are represented as mean ± SD (n >= 3). Significance was analyzed using Student's *t* test (*$p < 0.05$; ***$p < 0.001$).

(C) Mutually exclusive index of the H3K4me3-H3K36me3 and H3K27me3-H3K36me3 modifications in 2-cell stage NT embryos. A dashed line with an index equal to 1.0 indicates that no exclusive effects exist.

(D) Experimental schematic for the *Setd2* mRNA injection treatment. *Setd2* mRNA is injected into the reconstructed MII oocytes followed by activation. Two-cell stage NT embryos were collected for NChIP-seq for H3K36me3, H3K4me3 and H3K27me3, and Smart-seq2 for transcriptome profiling.

(E) Bar plot displays *Setd2* expression levels in 2-cell stage NT embryos (NT 2-C), 2-cell stage NF embryos (NF 2-C) and 2-cell stage NT embryo with *Setd2* mRNA injection (*Setd2*OE, *Setd2* overexpression). The expression levels were evaluated using an averaged log2TPM. Significance was analyzed by using Student's *t* test (**$p < 0.01$, ****$p < 0.0001$).

(F) Correlation of Smart-seq2 samples, H3K36me3 NChIP samples, H3K4me3 NChIP samples and H3K27me3 NChIP samples in 2-cell stage NT embryos with *Setd2* overexpression. Pearson's correlation coefficients of the replicates of Smart-seq2 samples were calculated using TPM of all RefSeq genes. Pearson's correlation coefficients of the replicates of H3K36me3, H3K4me3 and H3K27me3 NChIP samples were calculated using log2 (RPKM) of signal of all the 2-kb window regions.

Figure S10. Overexpression of *Setd2* reshaped patterns of multiple epigenetic marks and gene expression profiles in 2-cell stage NT embryos.

(A) Bar chart displays the proportion of the genome covered by H3K4me3 at promoter regions in NF 2-C, NT 2-C and NT 2-C Setd2OE embryos.

(B) Bar chart displays the proportion of the genome covered by H3K27me3 at promoter regions in NF 2-C, NT 2-C and NT 2-C Setd2OE embryos.

(C) Alluvial plot shows dynamics of H3K4me3 domains of the 4 categories (Broad, Medium, Narrow and Control) at promoter regions among NT 2-C, NF 2-C and NT 2-C *Setd2*OE samples.

(D) Alluvial plot shows dynamics of H3K27me3 domains at promoter regions among NT 2-C, NF 2-C and NT 2-C *Setd2*OE samples.

(E) PCA plot showing the genome-wide expression road map in NT and NF embryos. Color represents different developmental stages and shape represents types of samples.

(F) Violin plots demonstrating H3K4me3 domain length (the H3K4me3 Domain Length panel), H3K27me3 enrichment (the H3K27me3 panel) at ZGA gene promoters, and expression levels of ZGA genes (the RNA panel) in each cluster. Alluvial plots (the Chromatin State panel) illustrating the dynamics of H3K4me3-only, H3K27me3-only, bivalent and unmarked genes in all ZGA genes among NT 2-C, NF 2-C and NT 2-C *Setd2*OE samples. Significance analyzed using Student's *t* test (ns: not significant, *$p < 0.05$, **$p < 0.01$, ***$p < 0.001$, ****$p < 0.0001$).

Supplementary Table legends

Table S1. A summary of quality information of ChIP-seq data and Smart-seq2 data.

Table S2. A summary of correlation of replicates in each stage of NT embryos and CC.

Table S3. A summary of chromatin categories of genes.

Table S4. A summary of H3K4me3 domain width at promoter regions in each sample.

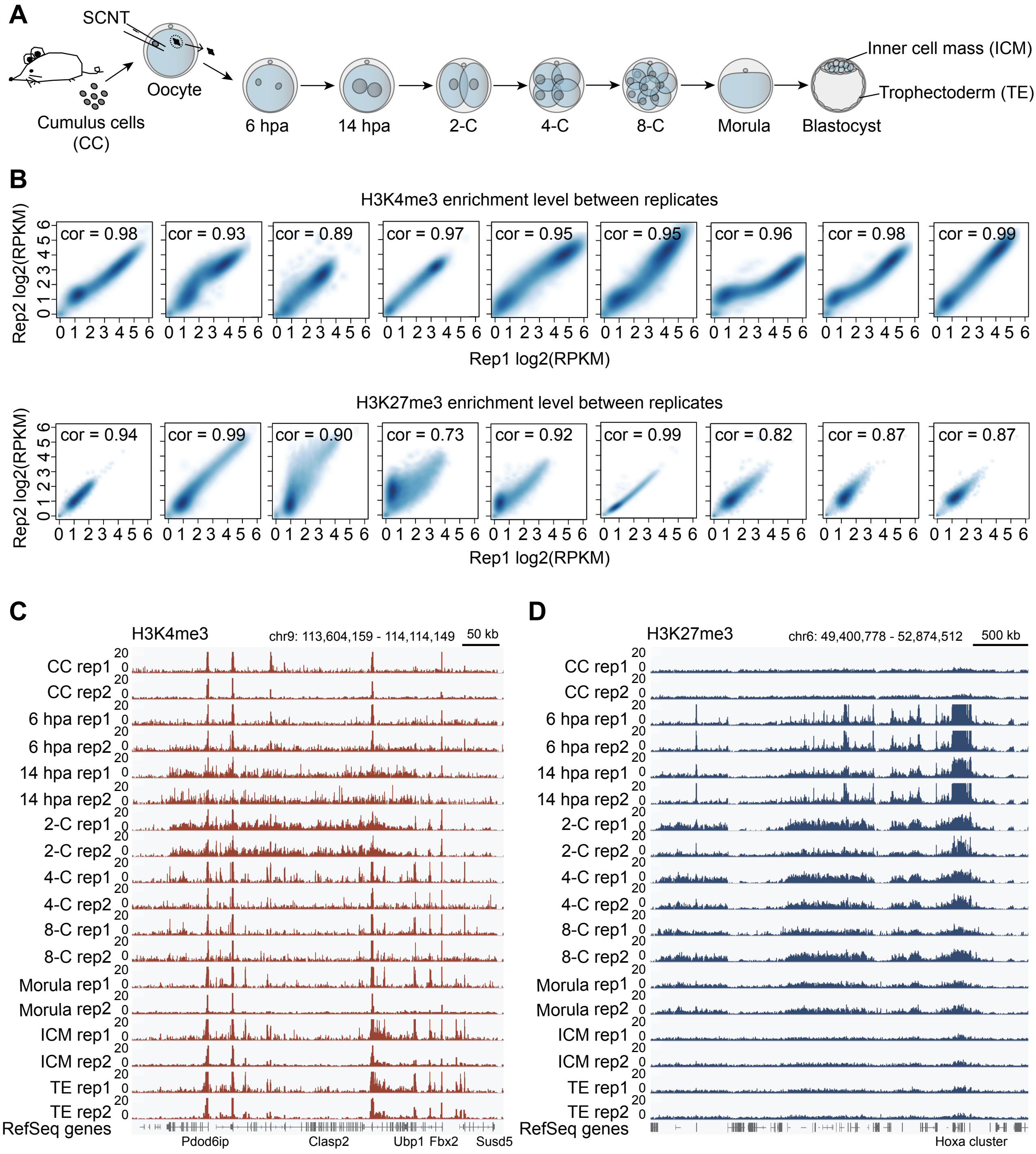
A
SCNT
Oocyte
Cumulus cells (CC)
6 hpa
14 hpa
2-C
4-C
8-C
Morula
Blastocyst
Inner cell mass (ICM)
Trophectoderm (TE)
B
H3K4me3 enrichment level between replicates
cor = 0.98
cor = 0.93
cor = 0.89
cor = 0.97
cor = 0.95
cor = 0.95
cor = 0.96
cor = 0.98
cor = 0.99
Rep2 log2(RPKM)
Rep1 log2(RPKM)
H3K27me3 enrichment level between replicates
cor = 0.94
cor = 0.99
cor = 0.90
cor = 0.73
cor = 0.92
cor = 0.99
cor = 0.82
cor = 0.87
cor = 0.87
Rep2 log2(RPKM)
Rep1 log2(RPKM)
C
H3K4me3
chr9: 113,604,159 - 114,114,149
50 kb
CC rep1
CC rep2
6 hpa rep1
6 hpa rep2
14 hpa rep1
14 hpa rep2
2-C rep1
2-C rep2
4-C rep1
4-C rep2
8-C rep1
8-C rep2
Morula rep1
Morula rep2
ICM rep1
ICM rep2
TE rep1
TE rep2
RefSeq genes
Pdod6ip
Clasp2
Ubp1
Fbx2
Susd5
D
H3K27me3
chr6: 49,400,778 - 52,874,512
500 kb
CC rep1
CC rep2
6 hpa rep1
6 hpa rep2
14 hpa rep1
14 hpa rep2
2-C rep1
2-C rep2
4-C rep1
4-C rep2
8-C rep1
8-C rep2
Morula rep1
Morula rep2
ICM rep1
ICM rep2
TE rep1
TE rep2
RefSeq genes
Hoxa cluster

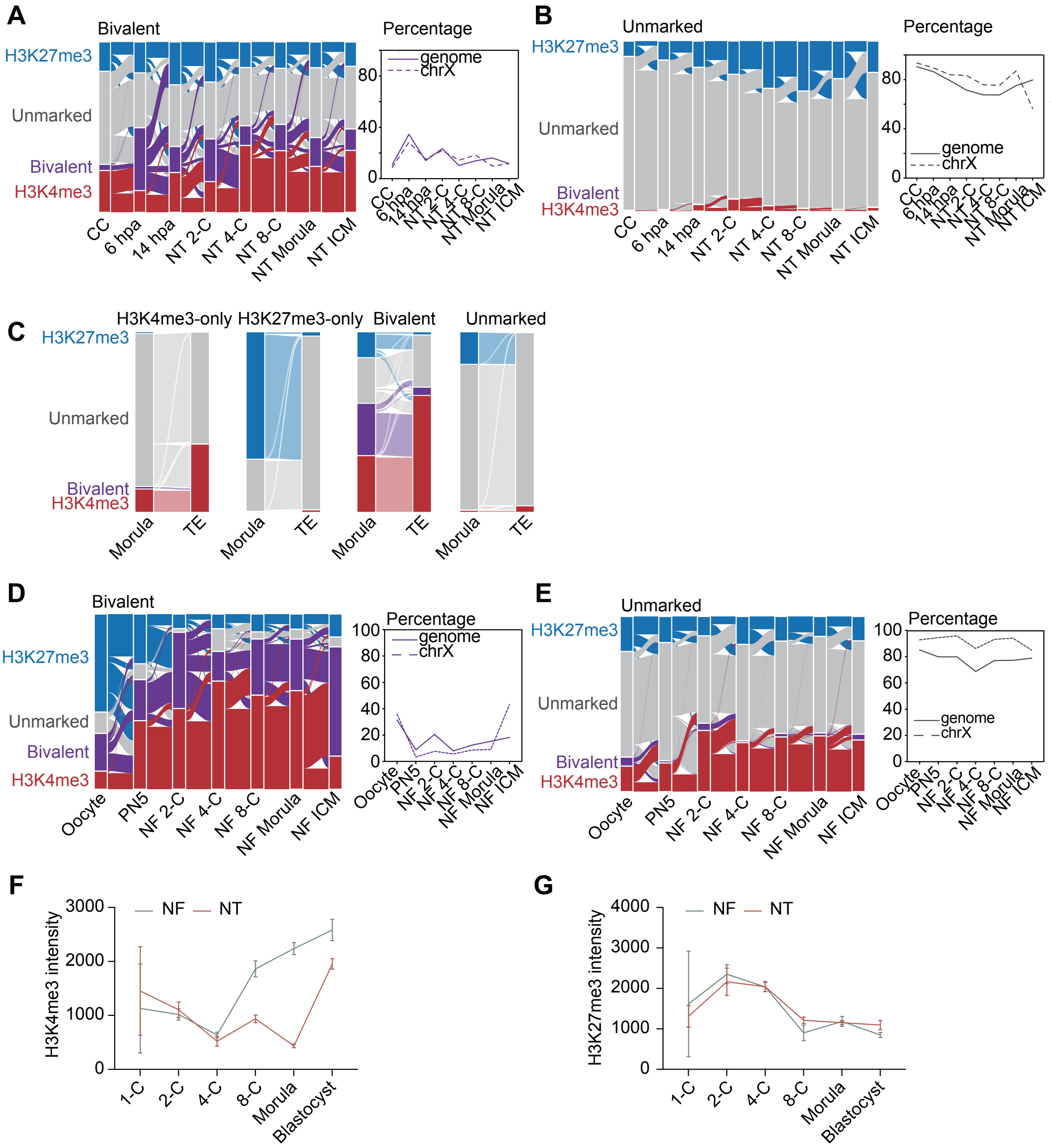

A
Bivalent
Percentage
H3K27me3
Unmarked
Bivalent
H3K4me3
genome
chrX
CC
6 hpa
14 hpa
NT 2-C
NT 4-C
NT 8-C
NT Morula
NT ICM
B
Unmarked
C
H3K4me3-only
H3K27me3-only
Morula
TE
D
Oocyte
PN5
NF 2-C
NF 4-C
NF 8-C
NF Morula
NF ICM
E
F
NF
NT
H3K4me3 intensity
1-C
2-C
4-C
8-C
Morula
Blastocyst
G
H3K27me3 intensity

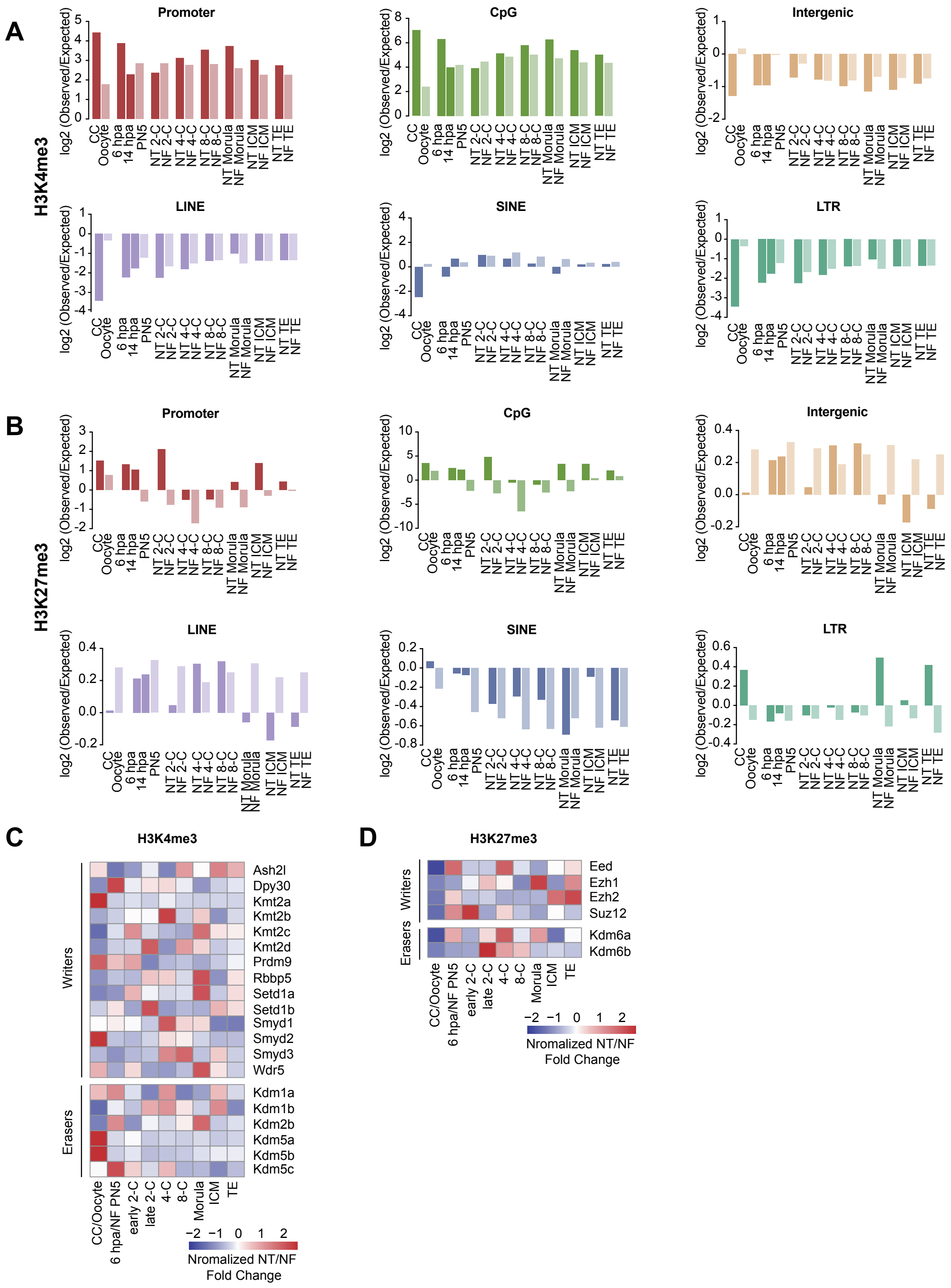
A
H3K4me3
Promoter
CpG
Intergenic
LINE
SINE
LTR
log2 (Observed/Expected)
CC
Oocyte
6 hpa
14 hpa
PN5
NT 2-C
NF 2-C
NT 4-C
NF 4-C
NT 8-C
NF 8-C
NT Morula
NF Morula
NT ICM
NF ICM
NT TE
NF TE
B
H3K27me3
C
H3K4me3
Writers
Erasers
Ash2l
Dpy30
Kmt2a
Kmt2b
Kmt2c
Kmt2d
Prdm9
Rbbp5
Setd1a
Setd1b
Smyd1
Smyd2
Smyd3
Wdr5
Kdm1a
Kdm1b
Kdm2b
Kdm5a
Kdm5b
Kdm5c
CC/Oocyte
6 hpa/NF PN5
early 2-C
late 2-C
4-C
8-C
Morula
ICM
TE
−2 −1 0 1 2
Nromalized NT/NF
Fold Change
D
H3K27me3
Eed
Ezh1
Ezh2
Suz12
Kdm6a
Kdm6b

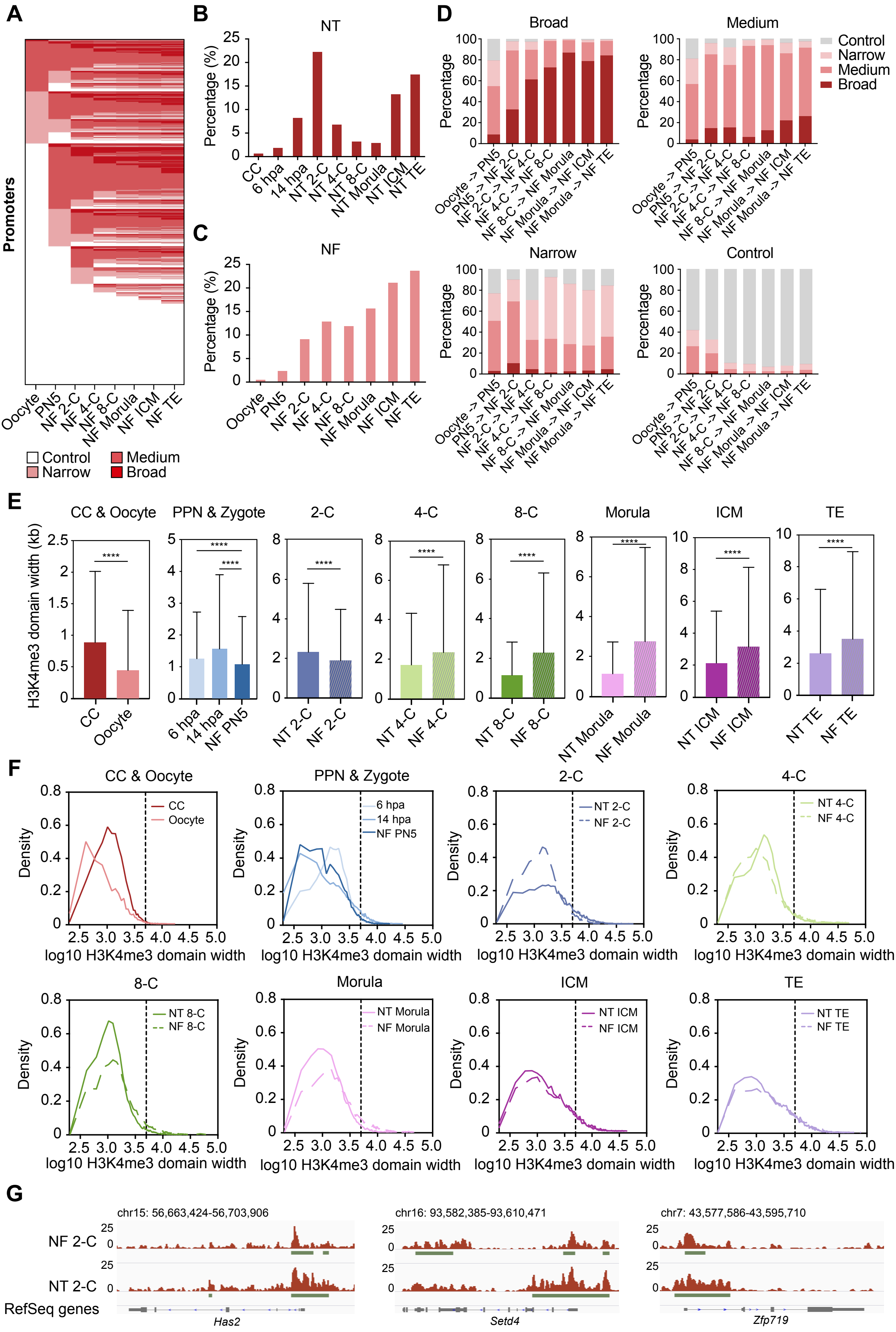
A
Promoters
Oocyte
PN5
NF 2-C
NF 4-C
NF 8-C
NF Morula
NF ICM
NF TE
Control
Narrow
Medium
Broad
B
NT
Percentage (%)
CC
6 hpa
14 hpa
NT 2-C
NT 4-C
NT 8-C
NT Morula
NT ICM
NT TE
C
NF
D
Broad
Medium
Narrow
Control
Percentage
Oocyte -> PN5
PN5 -> NF 2-C
NF 2-C -> NF 4-C
NF 4-C -> NF 8-C
NF 8-C -> NF Morula
NF Morula -> NF ICM
NF Morula -> NF TE
E
H3K4me3 domain width (kb)
CC & Oocyte
PPN & Zygote
2-C
4-C
8-C
Morula
ICM
TE
NF PN5
NF 2-C
NF 4-C
NF 8-C
NF Morula
NF ICM
NF TE
F
Density
log10 H3K4me3 domain width
G
chr15: 56,663,424-56,703,906
chr16: 93,582,385-93,610,471
chr7: 43,577,586-43,595,710
RefSeq genes
Has2
Setd4
Zfp719

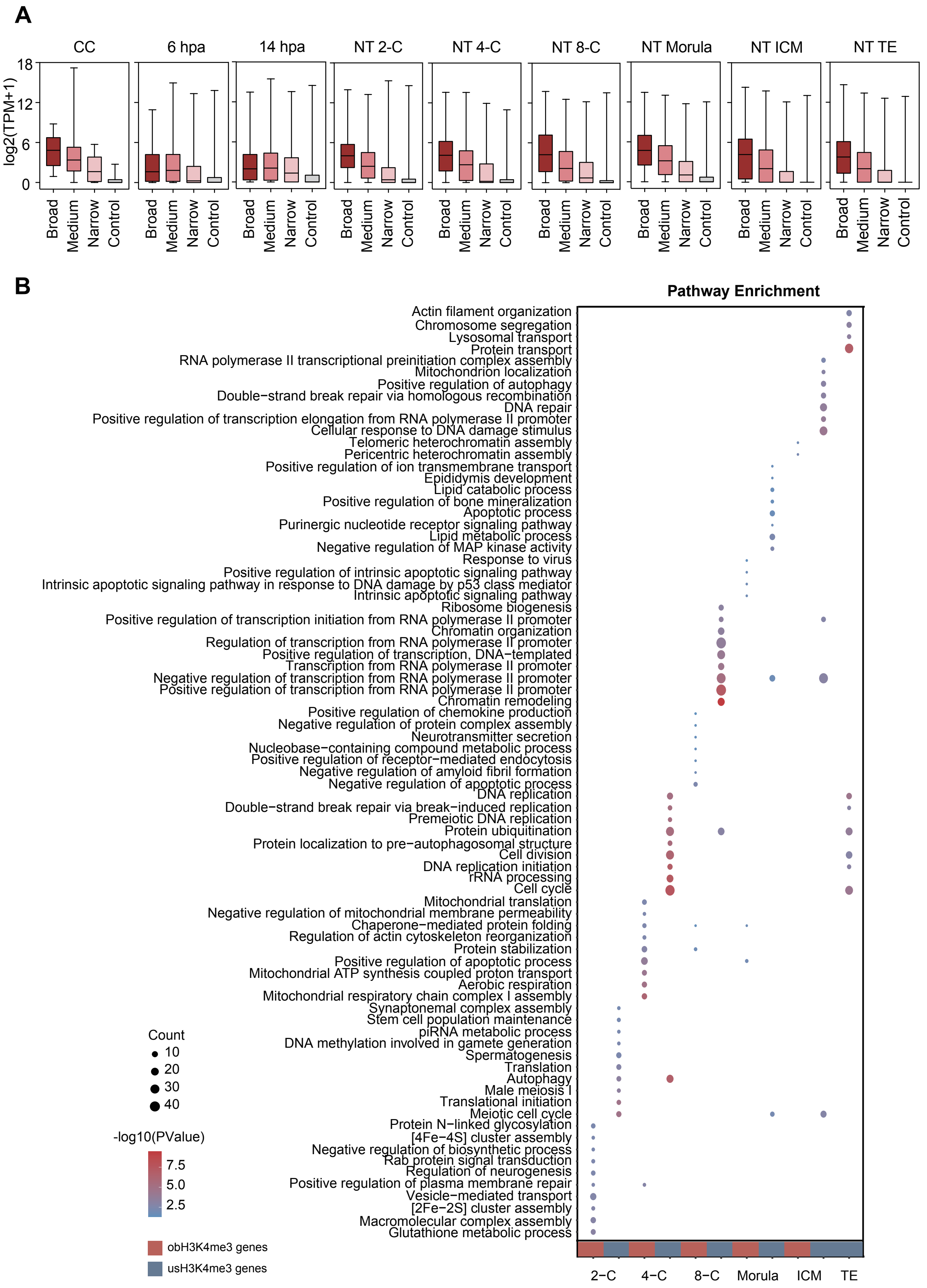

A
CC
6 hpa
14 hpa
NT 2-C
NT 4-C
NT 8-C
NT Morula
NT ICM
NT TE
log2(TPM+1)
18
12
6
0
Broad
Medium
Narrow
Control
B
Pathway Enrichment
Actin filament organization
Chromosome segregation
Lysosomal transport
Protein transport
RNA polymerase II transcriptional preinitiation complex assembly
Mitochondrion localization
Positive regulation of autophagy
Double−strand break repair via homologous recombination
DNA repair
Positive regulation of transcription elongation from RNA polymerase II promoter
Cellular response to DNA damage stimulus
Telomeric heterochromatin assembly
Pericentric heterochromatin assembly
Positive regulation of ion transmembrane transport
Epididymis development
Lipid catabolic process
Positive regulation of bone mineralization
Apoptotic process
Purinergic nucleotide receptor signaling pathway
Lipid metabolic process
Negative regulation of MAP kinase activity
Response to virus
Positive regulation of intrinsic apoptotic signaling pathway
Intrinsic apoptotic signaling pathway in response to DNA damage by p53 class mediator
Intrinsic apoptotic signaling pathway
Ribosome biogenesis
Positive regulation of transcription initiation from RNA polymerase II promoter
Chromatin organization
Regulation of transcription from RNA polymerase II promoter
Positive regulation of transcription, DNA−templated
Transcription from RNA polymerase II promoter
Negative regulation of transcription from RNA polymerase II promoter
Positive regulation of transcription from RNA polymerase II promoter
Chromatin remodeling
Positive regulation of chemokine production
Negative regulation of protein complex assembly
Neurotransmitter secretion
Nucleobase−containing compound metabolic process
Positive regulation of receptor−mediated endocytosis
Negative regulation of amyloid fibril formation
Negative regulation of apoptotic process
DNA replication
Double−strand break repair via break−induced replication
Premeiotic DNA replication
Protein ubiquitination
Protein localization to pre−autophagosomal structure
Cell division
DNA replication initiation
rRNA processing
Cell cycle
Mitochondrial translation
Negative regulation of mitochondrial membrane permeability
Chaperone−mediated protein folding
Regulation of actin cytoskeleton reorganization
Protein stabilization
Positive regulation of apoptotic process
Mitochondrial ATP synthesis coupled proton transport
Aerobic respiration
Mitochondrial respiratory chain complex I assembly
Synaptonemal complex assembly
Stem cell population maintenance
piRNA metabolic process
DNA methylation involved in gamete generation
Spermatogenesis
Translation
Autophagy
Male meiosis I
Translational initiation
Meiotic cell cycle
Protein N−linked glycosylation
[4Fe−4S] cluster assembly
Negative regulation of biosynthetic process
Rab protein signal transduction
Regulation of neurogenesis
Positive regulation of plasma membrane repair
Vesicle−mediated transport
[2Fe−2S] cluster assembly
Macromolecular complex assembly
Glutathione metabolic process
Count
10
20
30
40
-log10(PValue)
7.5
5.0
2.5
obH3K4me3 genes
usH3K4me3 genes
2−C
4−C
8−C
Morula
ICM
TE

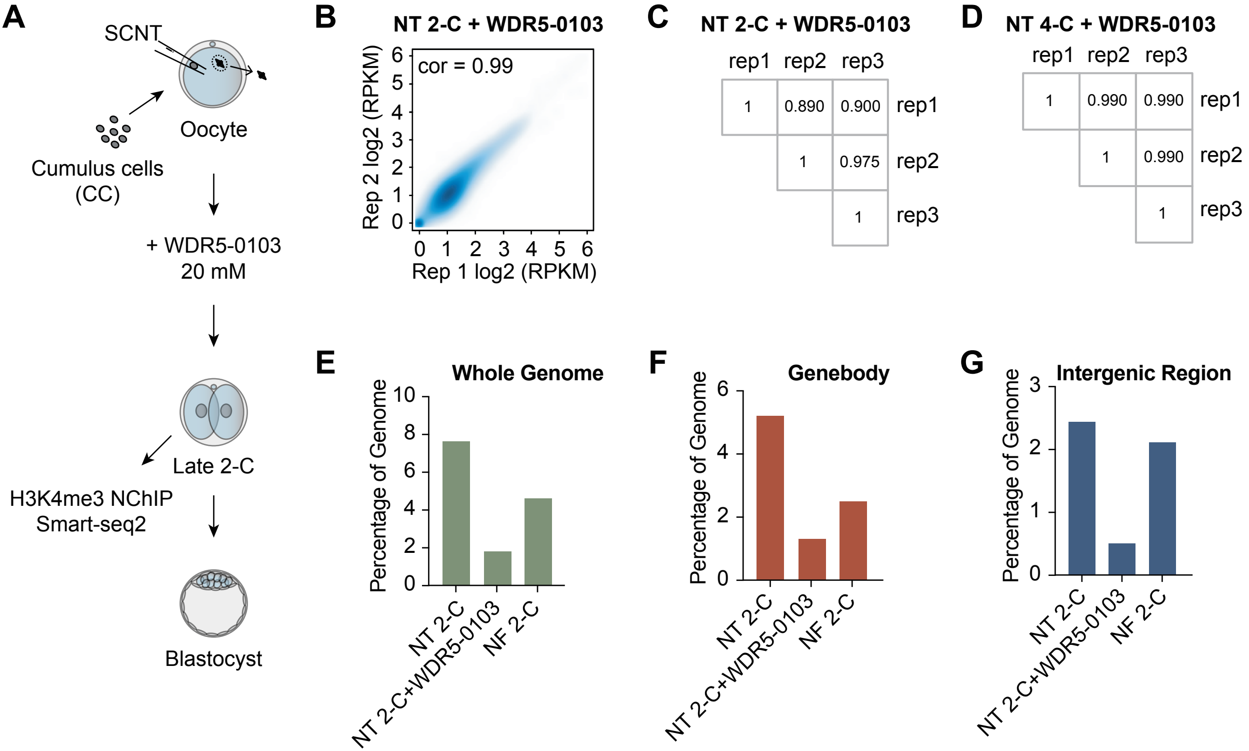
A
SCNT
Oocyte
Cumulus cells
(CC)
+ WDR5-0103
20 mM
Late 2-C
H3K4me3 NChIP
Smart-seq2
Blastocyst
B
NT 2-C + WDR5-0103
cor = 0.99
Rep 2 log2 (RPKM)
Rep 1 log2 (RPKM)
0 1 2 3 4 5 6
C
NT 2-C + WDR5-0103
rep1 rep2 rep3
1 0.890 0.900 rep1
1 0.975 rep2
1 rep3
D
NT 4-C + WDR5-0103
rep1 rep2 rep3
1 0.990 0.990 rep1
1 0.990 rep2
1 rep3
E
Whole Genome
Percentage of Genome
0 2 4 6 8 10
NT 2-C
NT 2-C+WDR5-0103
NF 2-C
F
Genebody
Percentage of Genome
0 2 4 6
NT 2-C
NT 2-C+WDR5-0103
NF 2-C
G
Intergenic Region
Percentage of Genome
0 1 2 3
NT 2-C
NT 2-C+WDR5-0103
NF 2-C

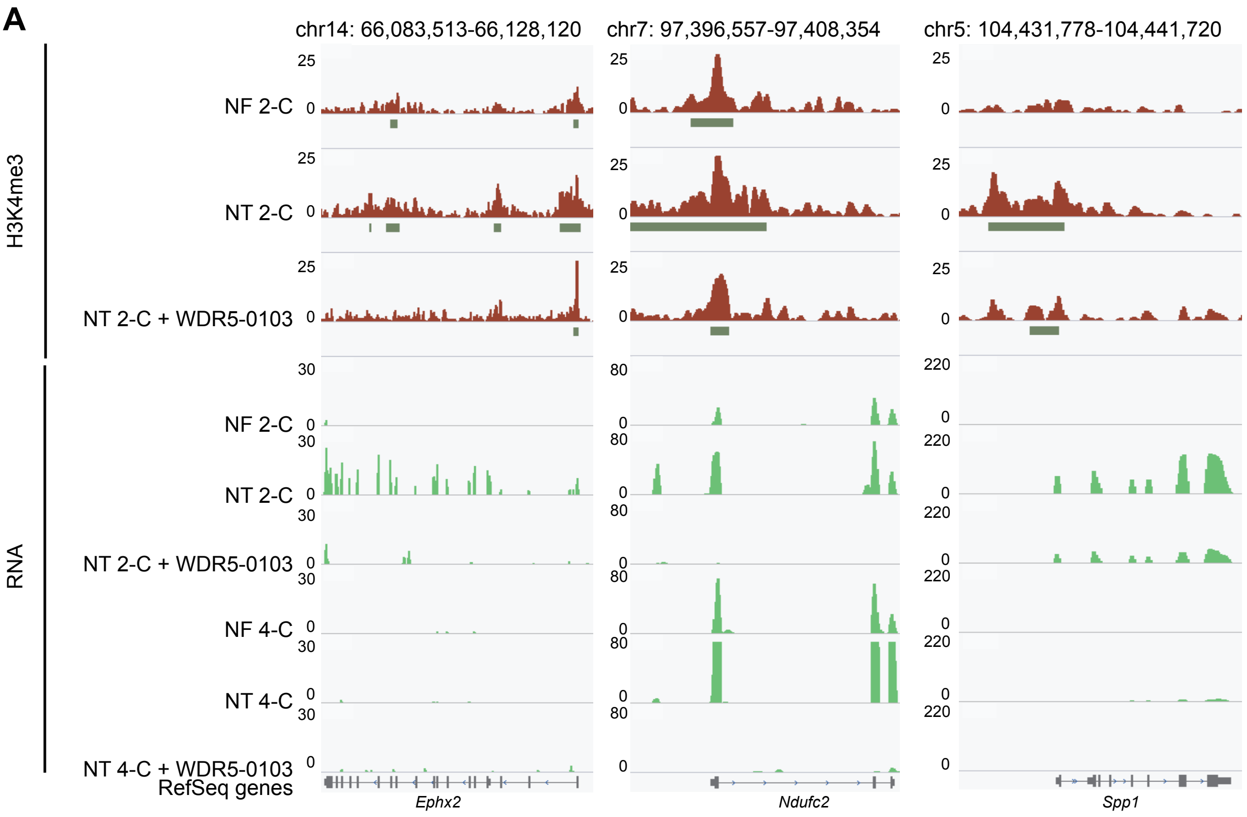
A
chr14: 66,083,513-66,128,120
chr7: 97,396,557-97,408,354
chr5: 104,431,778-104,441,720
H3K4me3
RNA
NF 2-C
NT 2-C
NT 2-C + WDR5-0103
NF 4-C
NT 4-C
NT 4-C + WDR5-0103
RefSeq genes
Ephx2
Ndufc2
Spp1

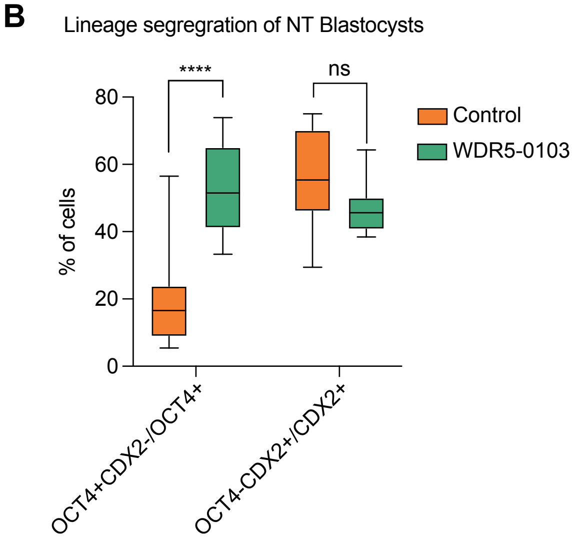
B
Lineage segregration of NT Blastocysts
% of cells
****
ns
Control
WDR5-0103
OCT4+CDX2-/OCT4+
OCT4-CDX2+/CDX2+

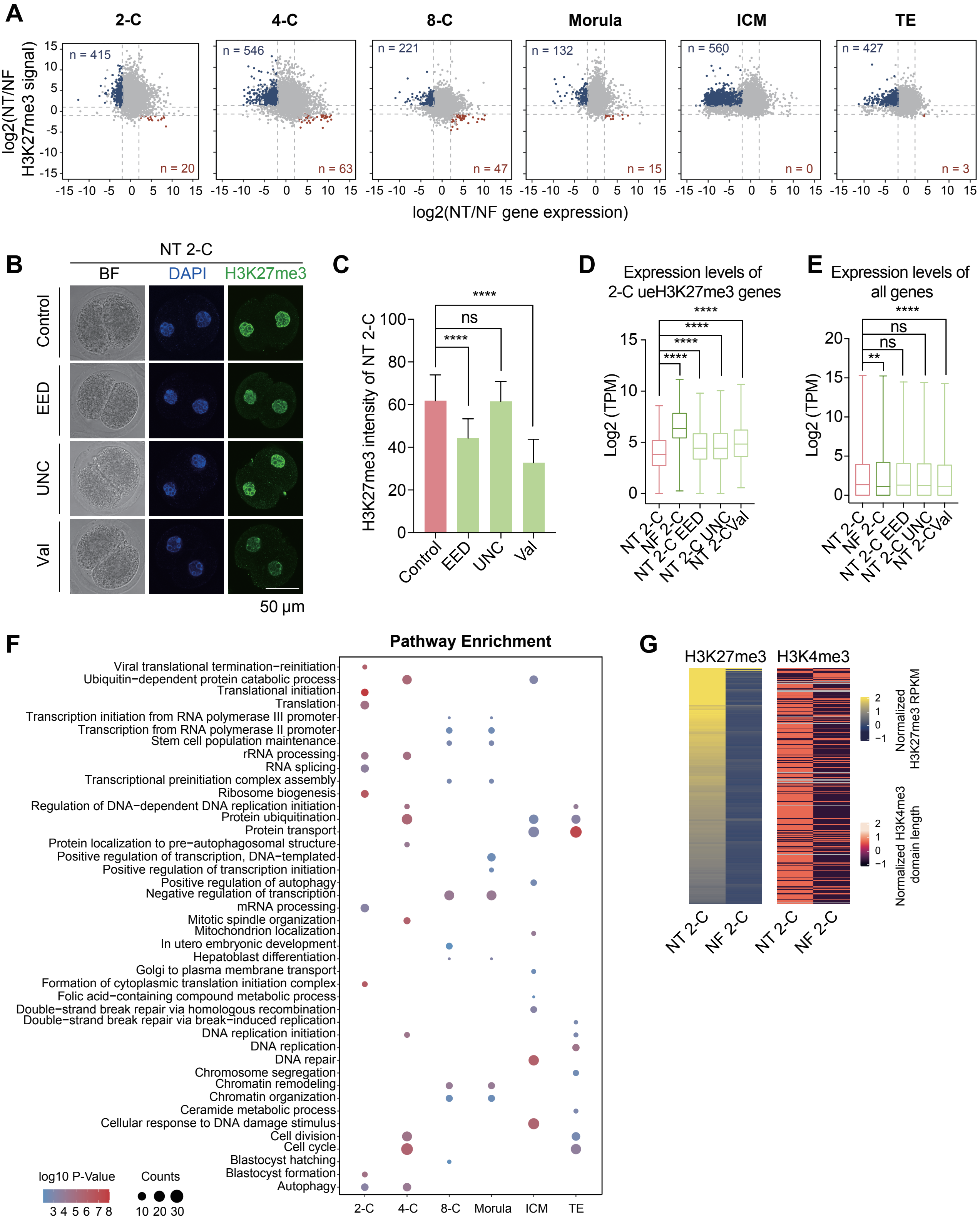
A
2-C
4-C
8-C
Morula
ICM
TE
log2(NT/NF H3K27me3 signal)
log2(NT/NF gene expression)
n = 415
n = 20
n = 546
n = 63
n = 221
n = 47
n = 132
n = 15
n = 560
n = 0
n = 427
n = 3
B
NT 2-C
BF
DAPI
H3K27me3
Control
EED
UNC
Val
50 μm
C
H3K27me3 intensity of NT 2-C
Control
EED
UNC
Val
****
ns
****
D
Expression levels of 2-C ueH3K27me3 genes
Log2 (TPM)
NT 2-C
NF 2-C
NT 2-C EED
NT 2-C UNC
NT 2-CVal
E
Expression levels of all genes
F
Pathway Enrichment
Viral translational termination−reinitiation
Ubiquitin−dependent protein catabolic process
Translational initiation
Translation
Transcription initiation from RNA polymerase III promoter
Transcription from RNA polymerase II promoter
Stem cell population maintenance
rRNA processing
RNA splicing
Transcriptional preinitiation complex assembly
Ribosome biogenesis
Regulation of DNA−dependent DNA replication initiation
Protein ubiquitination
Protein transport
Protein localization to pre−autophagosomal structure
Positive regulation of transcription, DNA−templated
Positive regulation of transcription initiation
Positive regulation of autophagy
Negative regulation of transcription
mRNA processing
Mitotic spindle organization
Mitochondrion localization
In utero embryonic development
Hepatoblast differentiation
Golgi to plasma membrane transport
Formation of cytoplasmic translation initiation complex
Folic acid−containing compound metabolic process
Double−strand break repair via homologous recombination
Double−strand break repair via break−induced replication
DNA replication initiation
DNA replication
DNA repair
Chromosome segregation
Chromatin remodeling
Chromatin organization
Ceramide metabolic process
Cellular response to DNA damage stimulus
Cell division
Cell cycle
Blastocyst hatching
Blastocyst formation
Autophagy
log10 P-Value
Counts
2-C
4-C
8-C
Morula
ICM
TE
G
H3K27me3
H3K4me3
Normalized H3K27me3 RPKM
Normalized H3K4me3 domain length
NT 2-C
NF 2-C
NT 2-C
NF 2-C

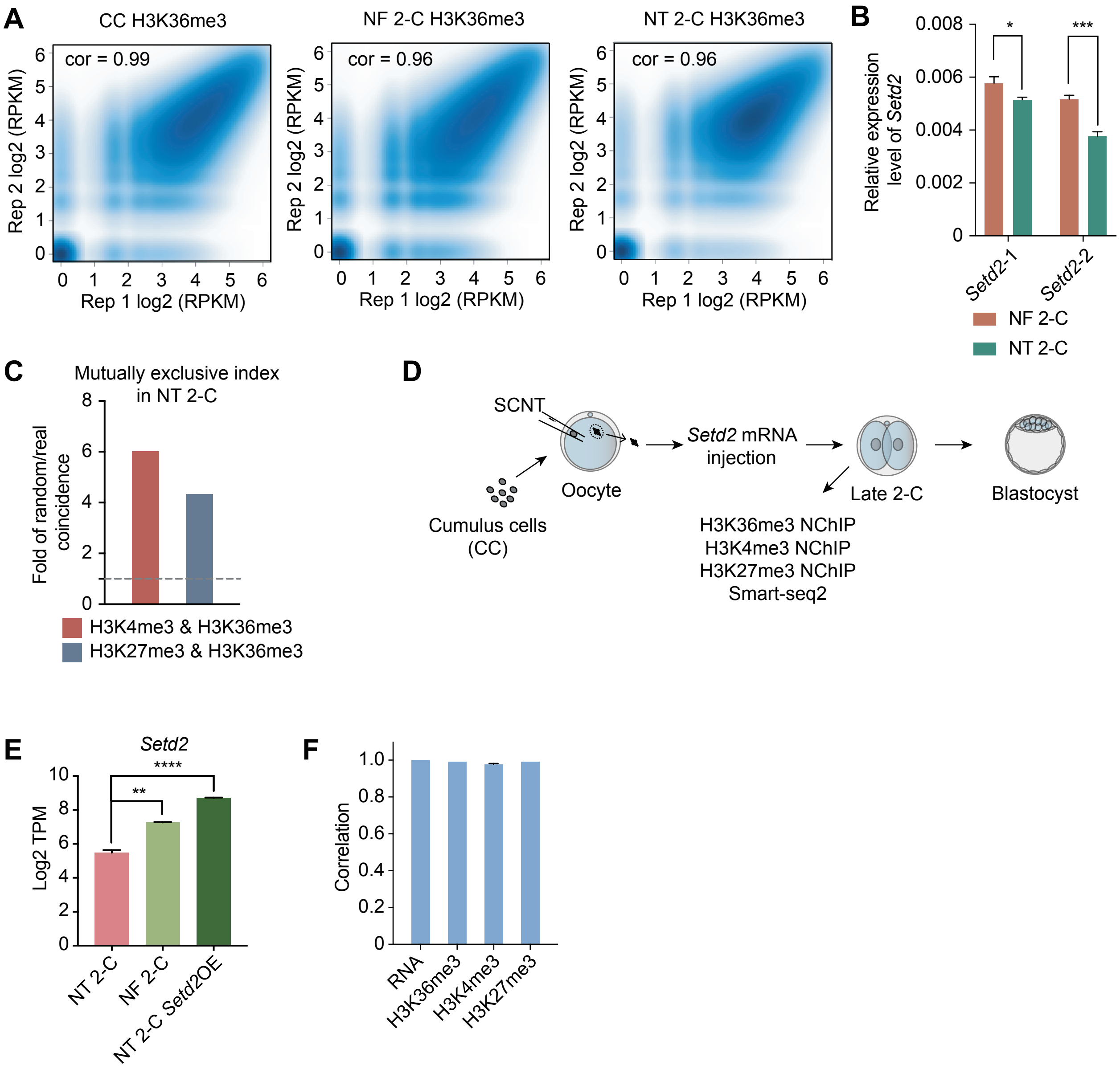
A
CC H3K36me3
cor = 0.99
NF 2-C H3K36me3
cor = 0.96
NT 2-C H3K36me3
cor = 0.96
Rep 2 log2 (RPKM)
Rep 1 log2 (RPKM)
B
Relative expression level of Setd2
Setd2-1
Setd2-2
NF 2-C
NT 2-C
C
Mutually exclusive index in NT 2-C
Fold of random/real coincidence
H3K4me3 & H3K36me3
H3K27me3 & H3K36me3
D
SCNT
Oocyte
Cumulus cells (CC)
Setd2 mRNA injection
Late 2-C
Blastocyst
H3K36me3 NChIP
H3K4me3 NChIP
H3K27me3 NChIP
Smart-seq2
E
Setd2
Log2 TPM
NT 2-C
NF 2-C
NT 2-C Setd2OE
F
Correlation
RNA
H3K36me3
H3K4me3
H3K27me3

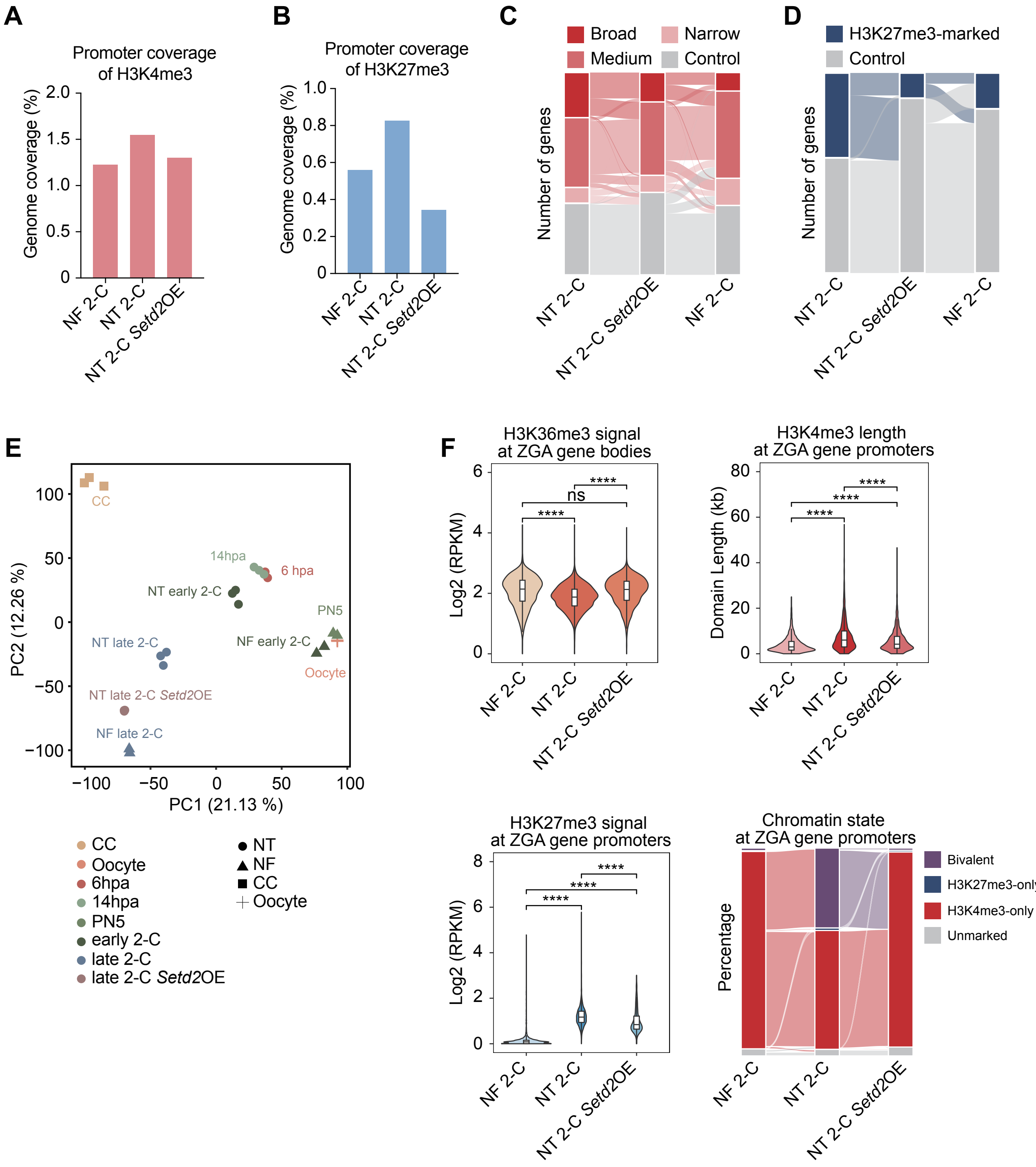

A
Promoter coverage of H3K4me3
Genome coverage (%)
2.0
1.5
1.0
0.5
0
NF 2-C
NT 2-C
NT 2-C Setd2OE
B
Promoter coverage of H3K27me3
Genome coverage (%)
1.0
0.8
0.6
0.4
0.2
0
NF 2-C
NT 2-C
NT 2-C Setd2OE
C
Broad
Narrow
Medium
Control
Number of genes
NT 2–C
NT 2–C Setd2OE
NF 2–C
D
H3K27me3-marked
Control
Number of genes
NT 2–C
NT 2–C Setd2OE
NF 2–C
E
CC
14hpa
6 hpa
NT early 2-C
PN5
NT late 2-C
NF early 2-C
Oocyte
NT late 2-C Setd2OE
NF late 2-C
PC2 (12.26 %)
PC1 (21.13 %)
100
50
0
−50
−100
−100
−50
0
50
100
CC
Oocyte
6hpa
14hpa
PN5
early 2-C
late 2-C
late 2-C Setd2OE
NT
NF
CC
Oocyte
F
H3K36me3 signal at ZGA gene bodies
Log2 (RPKM)
6
4
2
0
****
ns
****
NF 2-C
NT 2-C
NT 2-C Setd2OE
H3K4me3 length at ZGA gene promoters
Domain Length (kb)
80
60
40
20
0
****
****
****
NF 2-C
NT 2-C
NT 2-C Setd2OE
H3K27me3 signal at ZGA gene promoters
Log2 (RPKM)
8
6
4
2
0
****
****
****
NF 2-C
NT 2-C
NT 2-C Setd2OE
Chromatin state at ZGA gene promoters
Percentage
Bivalent
H3K27me3-only
H3K4me3-only
Unmarked
NF 2-C
NT 2-C
NT 2-C Setd2OE
